# Characterization of Three *Mycobacterium* spp. with Potential Use in Bioremediation by Genome Sequencing and Comparative Genomics

**DOI:** 10.1093/gbe/evv111

**Published:** 2015-06-16

**Authors:** Sarbashis Das, B.M. Fredrik Pettersson, Phani Rama Krishna Behra, Malavika Ramesh, Santanu Dasgupta, Alok Bhattacharya, Leif A. Kirsebom

**Affiliations:** ^1^Department of Cell and Molecular Biology, Uppsala University, Sweden; ^2^School of Life Sciences, Jawaharlal Nehru University, New Delhi, India

**Keywords:** genome sequencing, biodegradation, *Mycobacterium*, oxygenases, copper homeostasis

## Abstract

We provide the genome sequences of the type strains of the polychlorophenol-degrading *Mycobacterium chlorophenolicum* (DSM43826), the degrader of chlorinated aliphatics *Mycobacterium chubuense* (DSM44219) and *Mycobacterium obuense* (DSM44075) that has been tested for use in cancer immunotherapy. The genome sizes of *M. chlorophenolicum*, *M. chubuense*, and *M. obuense* are 6.93, 5.95, and 5.58 Mb with GC-contents of 68.4%, 69.2%, and 67.9%, respectively. Comparative genomic analysis revealed that 3,254 genes are common and we predicted approximately 250 genes acquired through horizontal gene transfer from different sources including proteobacteria. The data also showed that the biodegrading *Mycobacterium* spp. NBB4, also referred to as *M. chubuense* NBB4, is distantly related to the *M. chubuense* type strain and should be considered as a separate species, we suggest it to be named *Mycobacterium ethylenense* NBB4. Among different categories we identified genes with potential roles in: biodegradation of aromatic compounds and copper homeostasis. These are the first nonpathogenic *Mycobacterium* spp. found harboring genes involved in copper homeostasis. These findings would therefore provide insight into the role of this group of *Mycobacterium* spp. in bioremediation as well as the evolution of copper homeostasis within the *Mycobacterium* genus.

## Introduction

Bacteria of the genus *Mycobacterium* are acid fast, robust, and can inhabit various environmental reservoirs, for example, ground and tap water, soil, animals, and humans. This genus includes nonpathogenic environmental bacteria, opportunistic pathogens, and highly successful pathogens such as *Mycobacterium tuberculosis* (*Mtb*, the causative agent of tuberculosis). The diversity of ecological niches inhabited by *Mycobacterium* spp. demands widely varied life styles with different growth patterns and morphologies and ability to adapt to changes in the environment (Häggblom et al. 1994; Tortoli 2003, 2006; Primm et al. 2004; Vaerewijck et al. 2005; Falkinham 2009, 2015; Kazada et al. 2009; Whitman et al. 2012; Thoen et al. 2014). To understand the biology and versatility of *Mycobacterium* spp. we need to expand our knowledge about the genomic contents and their phenotypic expressions for different members of this genus.

*Mycobacterium chlorophenolicum*, *Mycobacterium chubuense*, and *Mycobacterium obuense* are classified as rapidly growing mycobacteria found in the same branch of 16S rRNA based phylogenetic trees. The first two are members of the *Mycobacterium sphagni* clade whereas *M. obuense* is positioned close but not adjacent to these two and belongs to the *Mycobacterium parafortuitum* clade (Apajalahti and Salkinoja-Salonen 1987; Miethling and Karlson 1996; McLellan et al. 2007; Whitman et al. 2012). These three species have been isolated from water, soil, and one isolate of *M. obuense* from the sputum of a patient. Strains related to these three species have the capacity to degrade different types of chlorinated pollutants. Hence they have the potential for use in bioremediation of contaminated soils (Whitman et al. 2012; Satsuma and Masuda 2012; see below).

*Mycobacterium chlorophenolicum* (originally referred to as *Rhodococcus chlorophenolicus*) was first isolated from a pentachlorophenol enrichment culture inoculated from chlorophenol-contaminated sediment from a lake in Finland (Briglia et al. 1994; Häggblom et al. 1994). It is a rapidly growing mesophilic *Mycobacterium* spp. with an optimal growth temperature of 28 °C and it produces yellow to orange colonies. It also shows “coccoid-to-rod-to-coccoid” morphological transitions during its growth cycle (Briglia et al. 1994; Häggblom et al. 1994). *Mycobacterium chlorophenolicum* can degrade polychlorinated phenol compounds such as pentachlorophenol (PCP; an environmental pollutant used in the past as a wood preservative agent [McLellan et al. 2007]) and its degradation products (Apajalahti and Salkinoja-Salonen 1987; Häggblom et al. 1994; Miethling and Karlson 1996). It has been used for in situ bioremediation of PCP-contaminated soils with some success (Briglia et al. 1994; Miethling and Karlson 1996). A recent study also shows that it can O-methylate tetrachlorobisphenol-A and tetrabromobisphenol-A, a brominated flame retardant used in consumer products, making them more lipophilic (George and Häggblom 2008).

*Mycobacterium chubuense* was first isolated from garden soil in Japan (Saito et al. 1977; Tsukamura and Mizuno 1977). It is rapidly growing, mesophilic, pigmented, and has rod- and-coccoid shaped cell morphologies and its position based on the 16S rRNA phylogenetic tree is close to *M. obuense* (98.5% sequence identity; Pitulle et al. 1992). Another isolate, originally referred to as *M. chubuense* NBB4 (and also *Mycobacterium* spp. NBB4, see below) has the potential for use in bioremediation. It is able to degrade chlorinated aliphatic compounds such as vinyl chloride and 1,2-dichloroethane, both intermediates in the production of polyvinyl chloride. In addition, a very broad range of hydrocarbons can promote its growth and the number of genes encoding mono-oxygenases is unusually high in this strain (Coleman et al. 2006; Le and Coleman 2011) as revealed by the complete genome sequence that was recently made available (acc no NC_018027.1).

*Mycobacterium obuense* was originally isolated from the sputum of a Japanese patient but it has also been isolated from soil samples and is not considered to be associated with any disease (Tsukamura and Mizuno 1971; Whitman et al. 2012). It has been described as a rod-shaped, rapidly growing bacterium that forms pigmented colonies. Recent data demonstrate that *M. obuense* too has the capacity, albeit limited, to reductively dechlorinate the insecticide methoxychlor that is used as an alternative to dichlorodiphenyltrichloro-ethane (DDT) (Masuda et al. 2012). *Mycobacterium obuense* as well as *M. chubuense* have been suggested as a stimulant of the immune system in bladder cancer therapeutics (Yuksel et al. 2011), and *M. obuense* is being evaluated in clinical trials for use in immunotherapy of several types of cancer (Fowler et al. 2011). Similar to *M. chlorophenolicum* and *M. chubuense*, *M. obuense* also has rod- andcoccoid-shaped cell morphologies (Saito et al. 1977). Moreover, both *M. obuense* and *M. chubuense* have been shown to be motile on agar surfaces (Agustí et al. 2008).

We decided to sequence the genomes of the type strains, *M. chlorophenolicum* DSM43826, *M. chubuense* DSM44219, and *M. obuense* DSM44075 (hereafter referred to as *Mchlo*DSM, *Mchu*DSM, and *Mobu*DSM, respectively), and to undertake comparative genomic analysis in order to understand how some of the characteristics of these genomes with respect to genome size, common and unique genes, horizontal gene transfer (HGT), and codon usage might be manifested as phenotypic differences. That these three *Mycobacterium* spp. do change their cell shape during cultivation (Saito et al. 1977; Häggblom et al. 1994) made such studies relevant to our interest in mechanisms of morphological changes seen in *Mycobacterium* spp. (Ghosh et al. 2009). We were also interested in understanding the evolutionary relationship between these *Mycobacterium* spp. and the *Mycobacterium* spp. NBB4 strain (hereafter referred to as *Myc*NBB4), in particular the relation between this strain and the *Mchu*DSM type strain. Here, we report a first analysis of their draft genomes. Interestingly, our data reveal that the *Mchu*DSM type strain is phylogenetically closer to *Mchlo*DSM than it is to the *Myc*NBB4 strain. This raises an important question about whether *Mchu*DSM and *Myc*NBB4 (also referred to as *M. chubuense* NBB4) belong to the same species*.* We also provide data showing the presence of putative genes encoding for oxygenases in all four species as well as for proteins involved in copper homeostasis in *Mchlo*DSM and in *Myc*NBB4.

## Materials and Methods

### Strains

The *M. chlorophenolicum* DSM43826 (*Mchlo*DSM), *M. chubuense* DSM44219 (*Mchu*DSM), and *M. obuense* DSM44075 (*Mobu*DSM) type strains were obtained from the Deutsche Sammlung von Mikroorganismen und Zellkulturen in Germany and grown under conditions as recommended by the supplier.

### Cultivation and DNA Isolation

Aliquots of the *Mchlo*DSM, *Mchu*DSM, and *Mobu*DSM type strains were taken from −80 °C stocks, plated on Middlebrook 7H10 media and incubated at 30 °C (*Mchlo*DSM) and 37 °C (*Mchu*DSM and *Mobu*DSM) under aerobic conditions. Extraction of DNA from these cultures and sequencing of the 16S rRNA genes after polymerase chain reaction amplification were consistent with the cultures being free from contaminations. Genomic DNA was isolated as previously described (Pettersson et al. 2014).

### Genome Sequencing, Assembly, and Annotation

Whole-genome sequencing was performed at the SNP&SEQ Technology Platform of Uppsala University on a HiSeq2000 (Illumina) platform.

The genomes of the *Mchlo*DSM, *Mchu*DSM, and *Mobu*DSM type strains were sequenced at coverage of 157×, 482×, and 285×, respectively. These genomes were assembled with the A5 assembly pipeline (Tritt et al. 2012). The A5-pipeline included the quality filtering of the reads, error correction, scaffolding, and gap filling steps. Genome alignment and reordering of the scaffolds was done using the Mauve program (Darling et al. 2004) and plotted with the R-package genoPlotR (Guy et al. 2010). rRNA and tRNA genes were identified using the RNAmmer (Lagesen et al. 2007) and tRNAScan-SE (Lowe and Eddy 1997) programs, respectively.

To predict the presence of plasmids, scaffolds were aligned with the *Myc*NBB4 complete genome (chromosome; acc no NC_018027.1). Scaffolds that did not align with the *Myc*NBB4 genome were subjected to BLAST using the NCBI plasmid database. We considered that scaffolds originated from plasmids if more than 90% of the scaffold sequence aligned with the plasmid database. Prophage sequences were predicted using the PHAST server (Zhou et al. 2011).

Identification and annotation of coding sequences (CDS) was done using both the Prokka software (version 1.0.9) (Seemann 2014) and the RAST server (http://rast.nmpdr.org/, last accessed May 5, 2015; Aziz et al. 2008). Functional classification was done using the RAST subsystem classification that uses data both from “The Project to Annotate 1000 genomes” and a collection of protein families referred to as FIGfams. Finally, the listed CDS are those that were predicted by both the Prokka and the RAST server. This annotation program also predicted genes encoding transposases and insertion sequence (IS) elements.

### Phylogenetic Analysis Based on Single and Multiple Genes

The sequences of the 16S ribosomal RNA genes (and *rpoB*, *dprE1*, and *rnpB*) were extracted from the draft *Mchlo*DSM, *Mchu*DSM, and *Mobu*DSM genomes and the publicly available *Myc*NBB4 genome (acc no NC_018027.1). The homologous sequences of these genes present in other *Mycobacterium* spp. including *Myc*NBB4 were downloaded from the NCBI database and aligned using the MAFFT (version 5) software (Katoh et al. 2005). Phylogenetic trees based on the multiple sequence alignment were computed using the FastTree software (Price et al. 2009) with 1,000 cycles of bootstrapping and the figures were generated with the FigTree software (http://tree.bio.ed.ac.uk/software/figtree/).

### Average Nucleotide Identity and Core Gene Analysis

The average nucleotide identity (ANI) was calculated using the Jspecies tool (Richter and Rosselló-Móra 2009) based on the sequenced genomes to identify whether those belonged to the same species or not. Core gene analysis was performed on the translated protein sequences of all predicted CDS. Protein sequences were subjected to “all-versus-all” BLAST and homologous sequences (referred to as “core genes”) were identified using PanOct with an identity of 45% and query coverage of 65% (Fouts et al. 2012).

### Horizontal Gene Transfer

Horizontally transferred genes were predicted based on BLAST best-hit approach using the newly available HGTector software, which follows a hybrid between “BLAST-based” and phylogenetic approaches (Zhu et al. 2014). This tool distributes the genes on the basis of the best BLAST hit into predefined hierarchical evolutionary categories: self, close, and distal based on NCBI taxonomy (as of July 2014). Genes that fall in the category “distal” are classified as putative HGT genes. We used the following stringent criteria: e-value set at <1e-100 for the BLAST hits, self = *Mycobacterium* (taxonomic_id 1763) and close = *Actinomycetales* (taxonomic_id 2037) groups (Zhu et al. 2014). Furthermore, common and unique putative horizontally transferred genes among the four genomes were identified using BLASTp with percentage identity of 45% and query coverage of 70%.

### Codon Usage Analysis

Relative synonymous codon usage analysis was done on nucleotide sequence of all the predicted genes and HGT genes in *Mchlo*DSM, *Mchu*DSM, *Mobu*DSM, and *Myc*NBB4 using the CodonW software (Peden 1999).

### Accession Numbers

The genome sequences have been deposited at GenBank/DDBJ/EMBL under the following accession numbers: JYNL00000000 *Mycobacterium chlorophenolicum* DSM43826, JYNX00000000 *Mycobacterium chubuense* DSM44219, and JYNU00000000 *Mycobacterium obuense* DSM44075.

## Results

### Genome Assembly, Alignment, Annotation, and Overall Description

**Fig. 1.—:**
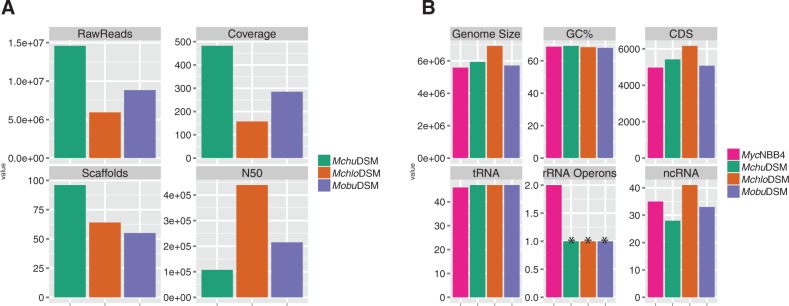
Genome assemblies and annotations. (*A*) Barplots showing number of raw reads, read coverage, number of scaffolds, and assembly quality (N50) for the three genomes represented by different colors as indicated. (*B*) Bar plots represent genome size, GC-content in %, number of tRNA genes, number of CDS, number of rRNA operons and noncoding RNA (ncRNA) for the four genomes represented by different color codes. Bars marked with * indicate that these genomes contains one complete and one partial rRNA operon.

The draft genome sequences of the *Mchlo*DSM, *Mchu*DSM, and *Mobu*DSM type strains were based on 59, 95, and 55 scaffolds (fig. 1*A*; supplementary figs. S1*A*, *C*, *E*, and *F*, Supplementary Material online) and their genome sizes were calculated to be 6,925,482 (*Mchlo*DSM), 5,945,132 (*Mchu*DSM), and 5,576,960 (*Mobu*DSM) base pairs, respectively (fig. 1*B*). As expected for mycobacterial species the GC-contents were high ranging from 67.9% to 69.2% (fig. 1*B*; Goodfellow et al. 2012; see also below). The total number of predicted CDS was found to be highest in *Mchlo*DSM, which correlates with its larger genome size (fig. 1*B*). Recently, the complete genome of the *Myc*NBB4 strain was released (acc no NC_018027.1). We therefore included the *Myc*NBB4 genome data in our analysis. Comparison of *Myc*NBB4 and *Mchu*DSM suggested that their genome sizes differ: the complete *Myc*NBB4 genome is approximately 0.4 Mb smaller than the *Mchu*DSM genome.

The presence of known plasmid sequences were identified in the *Mchlo*DSM and *Mobu*DSM draft genomes while no sequence of plasmid origin could be detected for *Mchu*DSM (see Materials and Methods). For *Mchlo*DSM, 454,038 bp in nine scaffolds were identified (supplementary fig. S1*B*, Supplementary Material online) and sequence alignment suggested that *Mchlo*DSM contains plasmid fragments of different origins similar to: 1) the *Mchu*NBB4 pMYCCH.01 (acc. no NC_018022.1), 2) the *M. gilvum* Spyr1 plasmid (acc. no NC_014811.1), and 3) *Mycobacterium smegmatis* JS623 pMYCSM02 (acc. no NC_019958.1). For *Mobu*DSM, 133,713 bp of plasmid origin located on three scaffolds were identified (supplementary fig. S1*D*, Supplementary Material online) and showed greater than 90% identity with sequences of pMKMS01 (acc no NC_008703.1), which is present in *Mycobacterium* spp. KMS. For *Mchlo*DSM, the plasmid fragments were predicted to carry 502 putative genes and 59% of these were annotated as hypothetical proteins. For functional annotation see supplementary table S1, Supplementary Material online.

**Fig. 2.—:**
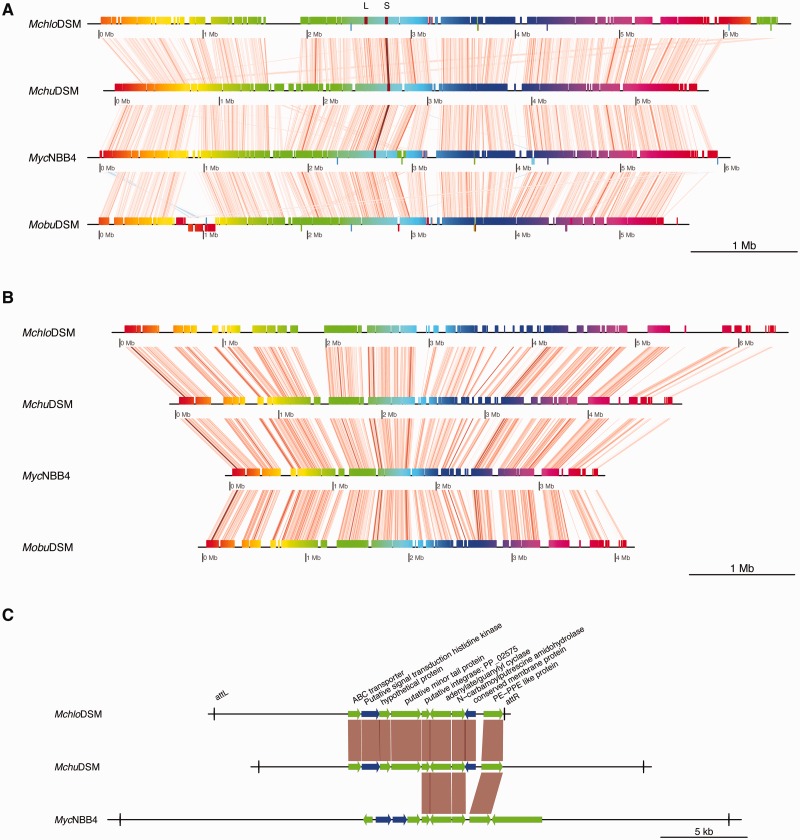
Whole genome and CDS alignment of the four genomes. (*A*) Whole genome alignment and (*B*) complete CDS alignment for the four *Mycobacterium* spp. as indicated. Each of the colored horizontal lines represents one genome and the vertical bars represent homologous regions. Light brown to dark vertical lines represent small to large homologous fragments and diagonal lines represent genomic rearrangements whereas red blocks below the black line which is connected with the blue diagonal lines mark inversions. (*C*) Gene synteny plot of the conserved small (marked with S; the large prophage sequences in *Mchlo*DSM is marked with L) prophage sequence predicted in the *Mchlo*DSM, *Mchu*DSM, and *Myc*NBB4. Black horizontal lines represent prophage sequences in the respective genomes. Blue and green arrows indicate predicted CDS of bacterial and phage origin, respectively. Vertical lines represent the attachment sites.

One of the reasons of genome rearrangement is due to the presence of IS elements and the IS116/IS110/IS902-family (Moss et al. 1992; Kulakov et al. 1999) was identified to be present in the *Mchlo*DSM, *Mchu*DSM, and *Mobu*DSM genomes (the light brown diagonal lines in fig. 2*A* suggest genomic rearrangements involving IS elements). Moreover, in *Mchlo*DSM a total of 19 copies of genes encoding transposases were predicted and of these, seven were located on plasmid fragments. *Mchu*DSM and *Mobu* carry fewer copies one and five, respectively. We emphasize that due to the presence of repeated sequences such as IS elements hinder the assembly of genomes into one single scaffold.

Prophage sequences including attachment sites were predicted in *Mchlo*DSM, *Mchu*DSM, and *Myc*NBB4 but not in *Mobu*DSM. The *Mchlo*DSM genome carries two fragments, 25 and 17 kb covering 26 and 9 CDS, respectively (fig. 2*A* and supplementary fig. S1*A*, Supplementary Material online). The smaller fragment is conserved in *Mchu*DSM and partially conserved in *Myc*NBB4 and it was predicted to encode mainly phage proteins (fig. 2*C*). However, it also carries a gene encoding a protein belonging to the PE-PPE family of proteins, which are commonly present among mycobacteria such as *M. tuberculosis* (Cole et al. 1998). For genes predicted to be located on the large prophage fragment in *Mchlo*DSM see supplementary table S2, Supplementary Material online.

One complete and one partial ribosomal operon were identified in the three draft genomes whereas in the complete *Myc*NBB4 genome two complete ribosomal RNA operons are present. We did, however, detect the presence of partial sequences that corresponded to rRNA operons (including two genes encoding 5S rRNA) in all the three draft genomes. Moreover, for all three genomes the average read depth of the genomic region carrying ribosomal RNA genes was 2-fold higher compared with the rest of the scaffold (supplementary fig. S2, Supplementary Material online). Together this suggested that *Mchlo*DSM, *Mchu*DSM, and *Mobu*DSM also have two complete ribosomal RNA operons in keeping with what is known for other rapidly growing *Mycobacterium* spp. (Ji et al. 1994); however see also (Stadthagen-Gomez et al. 2008).

The transfer RNA genes were annotated using the tRNAScan-SE (Lowe and Eddy 1997) and the numbers are shown in figure 1*B*. We identified 47 tRNA genes in *Mchlo*DSM, *Mchu*DSM, and *Mobu*DSM suggesting that the numbers of functional tRNA isoacceptors were the same in these three species. In *Mchlo*DSM, we also detected a tRNA-CGA pseudogene. The different tRNA genes are scattered around the chromosomes at roughly the same positions relative to the “*oriC*” in all four species (supplementary figs. S1*A*, *C*, *E*, and *F*, Supplementary Material online; the position of *oriC* is inferred from the position of *dnaA* [and *dnaN*] and *rpmH* [Gao et al. 2013 and references therein]; note that in *Mobu*DSM the positioning of *rpmH* relative to *dnaA* is altered compared with the other three strains). Also comparing the positioning of the different tRNA isoacceptor genes revealed that the same isoacceptor genes cluster in a similar way in the four strains with just a few exceptions (supplementary figs. S1*A*, *C*, *E*, and *F*, Supplementary Material online). The complete list of tRNAs identified are shown in supplementary table S3, Supplementary Material online. Comparison with *Myc*NBB4, however, indicated that one tRNA gene was missing in this strain. Interestingly, this corresponded to a gene encoding a tRNA^Cys^ isoacceptor. (Note: many bacteria only have one gene encoding tRNA^Cys^ [http://trna.bioinf.uni-leipzig.de]; see Discussion). All the other three strains have two genes encoding cysteine tRNA, *cysT* and *cysU* (*cysU* is marked with a * on the *Mchlo*DSM, *Mchu*DSM, and *Mobu*DSM chromosomes; supplementary fig. S1*A*, *C*, and *E*, Supplementary Material online). The *cysU* gene is located near the tRNA^Leu^(CAA) isoacceptor gene in all three species between genes encoding an arabinose efflux permease family protein and a small multidrug resistance protein. Analysis of the gene synteny covering this region in all four strains revealed that in *Myc*NBB4 *cysU* is missing at this location, possibly due to a deletion event (supplementary fig. S3*A*, Supplementary Material online). Moreover, sequence alignment of *cysT* and *cysU* revealed differences in: the amino acid acceptor-stem, the D-stem/ loop, the anticodon stem, the variable loop, and the T-loop. This might indicate possible differences in the amino acid charging of these two tRNA^Cys^ isoacceptors. We also noted that the 3′-terminal CA sequence was not encoded in *cysU* suggesting that formation of the 3′CCA termini occurred posttranscriptionally possibly involving the enzyme nucleotidyl transferase (supplementary fig. S3*B*, Supplementary Material online; Martin et al. 2008). In this context, we would also like to emphasize that tRNA genes are implicated to be targets for integration of foreign DNA, for example, pathogenicity islands (for ref. see e.g., Hacker and Kaper 2000; Juhas et al. 2009).

### Whole Genome Alignment Revealed Homologous and Unique Genomic Regions

Whole genome (excluding the plasmid fragments) and CDS alignments of the newly assembled genomes were generated using Mauve (see Materials and Methods; fig. 2). As apparent from figure 2*A Mchlo*DSM harbors the highest number of unique regions and genes (949; see also below), which was expected since its genome size was larger compared with the other genomes (fig. 1*B*). Similar patterns were also observed by whole CDS alignment (see fig. 2*B*). The genome wide ANI for the *Mchlo*DSM and *Mchu*DSM type strains was higher (95.7%) compared with the ANI for *Mchu*DSM and *Myc*NBB4 (85%; supplementary fig. S4, Supplementary Material online). In fact, the ANI values comparing *Mchu*DSM with *Myc*NBB4 (85%) were very similar to the values comparing *Mchu*DSM with *Mobu*DSM (85.5%) (we emphasize that comparing *Mobu*DSM and *Myc*NBB4 resulted in an ANI value of 84.5%). Moreover, the *Mobu*DSM showed low and similar ANI values when compared with *Mchu*DSM and *Mchlo*DSM, 85.5% and 85.7%, respectively. The genomes were clustered based on the ANI values using hierarchical clustering and the result indicated that *Mchu*DSM is closer to *Mchlo*DSM than it is to *Myc*NBB4 (supplementary fig. S4, Supplementary Material online; see also below).

### *Mycobacterium chlorophenolicum* DSM43826 and *M. chubuense* DSM44219 Show High Numbers of Homologous Genes

**Fig. 3.—:**
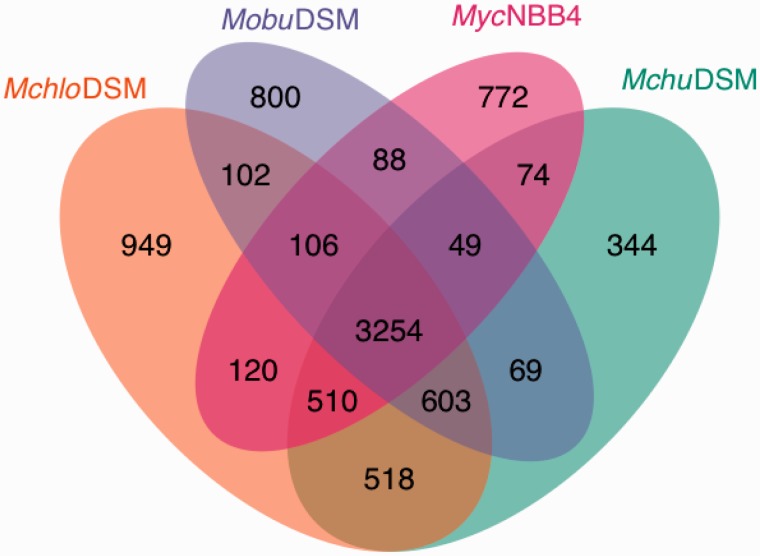
Venn diagram—presence of homologous and nonhomologous genes. The Venn diagram represents homologous and nonhomologous genes present in *Mchlo*DSM, *Mchu*DSM, *Mobu*DSM, and *Myc*NBB4. The Venn diagram was generated as outlined in Materials and Methods and the different mycobacterial strains are color coded as indicated.

Homologous and nonhomologous chromosomal genes were identified among the four mycobacterial strains using “BLASTp” with 45% identity and 70% query coverage and e-value 1e-05 cut offs. Relative to the *Mchu*DSM strain pairwise comparison of genes suggested that 90% of its genes are homologous to genes present in *Mchlo*DSM (supplementary fig. S5, Supplementary Material online; n*_MchuMchlo_*/ n*_Mchu_*_,_ where n*_MchuMchlo_* = 4,885 and n*_Mchu_* = 5,421). In contrast, only 78% (n*_MchuM__yc_*_NBB4_/ n*_M__yc_*_NBB4_, where n*_MchuM__yc_*_NBB4_ = 3,887 and n*_M__yc_*_NBB4_ = 4,973; fig. 3 and supplementary fig. S5, Supplementary Material online) of the *Myc*NBB4 genes have homologs that are present in *Mchu*DSM. Interestingly, 80% of the *Myc*NBB4 genes are homologous with genes present in the *Mchlo*DSM type strain and 70% with those in *Mobu*DSM. Moreover, comparative analysis of the homologous genes in all the four *Mycobacterium* spp. suggested that 3,254 homologs are present in all four strains. These genes are referred to as core genes (fig. 3).

*Mchlo*DSM has the highest number of unique genes (*n* = 949) among the four mycobacterial strains. The number of unique genes in the *Mchu*DSM and *Mobu*DSM type strains are lower 344 and 800, respectively. In contrast, the *Myc*NBB4 strain has a fairly large number of unique genes, almost 2-fold higher than that of the *Mchu*DSM type strain, even though its genome size is the smallest of these four mycobacterial strains (fig. 3). Together this again indicates that the *Mchu*DSM type strain is more distantly related to *Myc*NBB4 than it is to *Mchlo*DSM.

### Phylogenetic Analysis

**Fig. 4.—:**
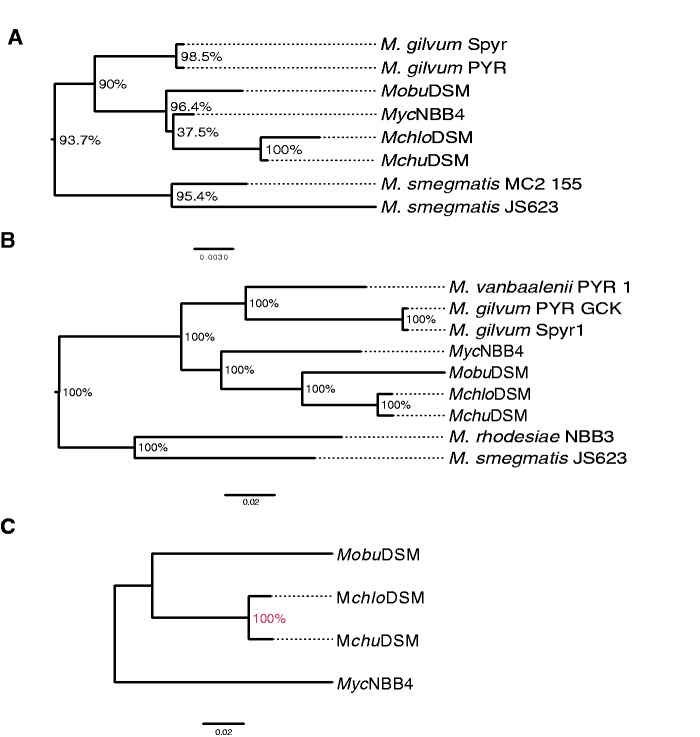
Phylogenetic analysis. Phylogenetic trees were generated based on (*A*) 16S rRNA complete gene sequences and (*B*) core genes in *Mycobacterium* spp., for details see main text. (*C*) A phylogenetic tree where we used the 3,268 homologous genes that were identified to be present in the *Mchlo*DSM, *Mchu*DSM, *Mobu*DSM, and *Myc*NBB4 genomes as indicated. Bootstrap values in percentage are shown at the common nodes.

We performed phylogenetic analysis of these four mycobacterial strains using a set of genes to understand their evolutionary positions with respect to other *Mycobacterium* spp. (see Materials and Methods). The genes selected for this analysis were: 1) the 16S rRNA and *rpoB* genes, which have been used extensively in phylogenetic analysis and 2) the *rnpB* and *dprE*1 genes, which have been used to a lesser extent but have been demonstrated to discriminate between different mycobacterial species as well as the 16S rRNA and *rpoB* genes, if not better (Incandela et al. 2013; Herrmann et al. 2014). Protein sequences were used for the analysis with *rpoB* and *dprE*1 whereas the DNA sequences were used for the other two. In addition, we used both the 3,254 core genes (protein sequence) that are present in all four mycobacterial strains (see above) and the 671 *Mycobacterium* core genes that corresponds to homologous genes present in all available *Mycobacterium* spp. deduced from complete genome sequences as indicated in figure 4 and supplementary figure S6, Supplementary Material online (see also supplementary table S4, Supplementary Material online).

The different phylogenetic trees were consistent with our current understanding of mycobacterial phylogeny. The results suggested that *Mchlo*DSM and *Mchu*DSM are the closest neighbors whereas the *Myc*NBB4 and *Mobu*DSM strains are more distantly related to both *Mchlo*DSM and *Mchu*DSM (fig. 4 and supplementary fig. S6, Supplementary Material online). This relationship also corroborate with the genomic distances derived from ANI values discussed above and raises the possibility that *Mchu*DSM (which belongs to the *M. sphagni* clade [Whitman et al. 2012]) and *Myc*NBB4 might belong to different clades.

### Functional Classification of Common and Unique Genes

**Fig. 5.—:**
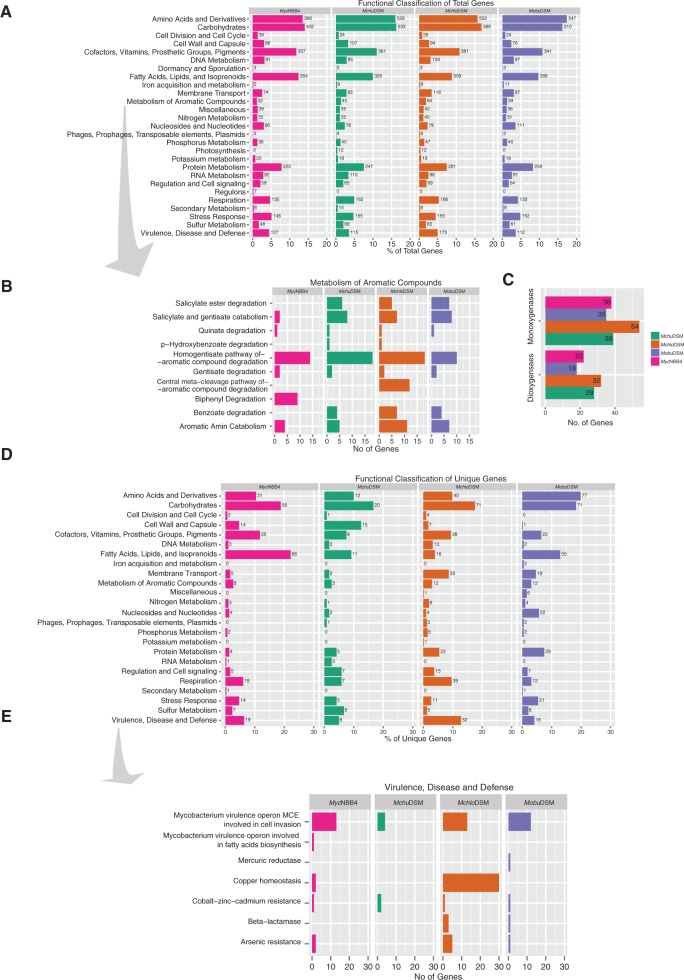
Functional classifications of total and unique genes. Bar plots representing functional classifications of genes in different categories: (*A*) Total predicted genes, (*B*) subclassification of genes that belong to the category metabolism of aromatic compounds, (*C*) number of genes encoding mono- and dioxygenases in the four *Mycobacterium* spp. as indicated, (*D*) unique genes in the four mycobacterial strains (*Mchlo*DSM, *Mchu*DSM, *Mobu*DSM, and *Myc*NBB4), and (*E*) subclassification of the unique genes that belong to the category “virulence, disease, and defense.” Different colors represent different genomes as indicated. In (*A*) and (*D*) the *x* axis represents percentage of total genes whereas in (*B*), (*C*), and (*E*) the *x* axis corresponds to the number of genes.

**Fig. 6.—:**
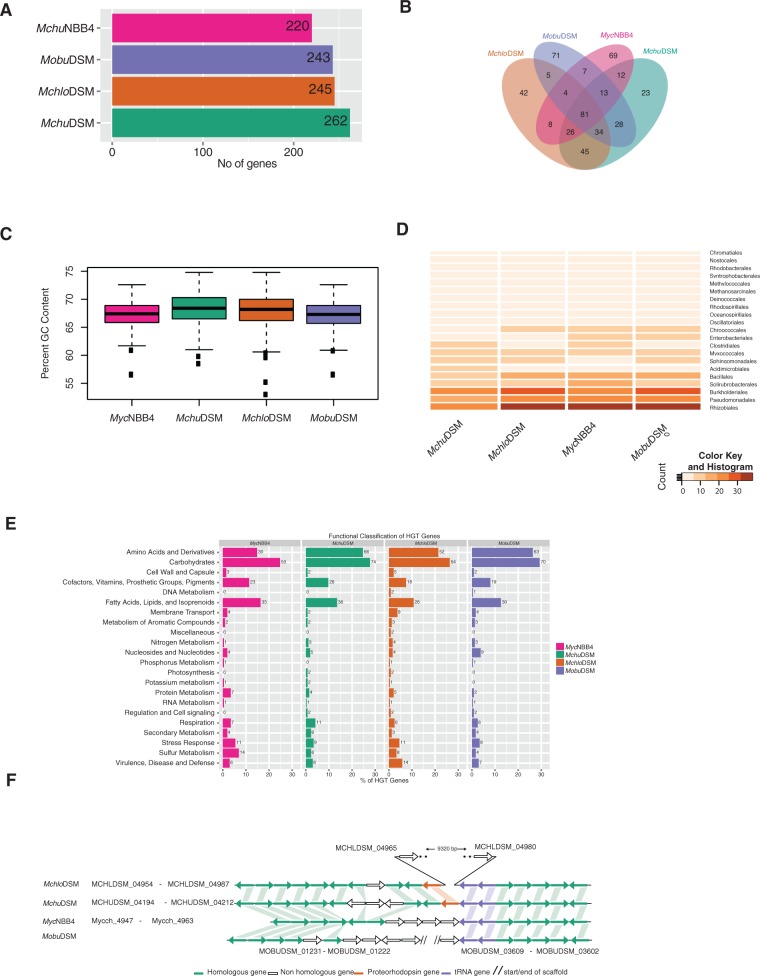
Identification and characterization of horizontally transferred genes. (*A*) Horizontal bar plots showing number of HGT-genes identified in the *Mchlo*DSM, *Mchu*DSM, *Mobu*DSM, and *Myc*NBB4 genomes. *x* axis represents number of HGT-genes and *y* axis shows the four genomes as indicated. (*B*) Venn diagram showing common and unique HGT-genes. (*C*) Box plot showing percentage GC-content of the HGT-genes in the *Mchlo*DSM, *Mchu*DSM, *Mobu*DSM, and *Myc*NBB4 genomes. *y* axis represents percentage GC-content. The horizontal lines represent the first (25%), second (50%), third (75%), and forth (100%) quartiles. The thick horizontal line in the middle of each colored box represents the median value and filled squares are the outliers. (*D*) Heat map showing the probable source of the HGT-genes (see also supplementary fig. S8*A*, Supplementary Material online). Color code: dark brown refers to high while light colors to fewer numbers of genes. (*E*) Functional classification of the HGT-genes using subsystem classifications. *x* axis represents number of the HGT-genes in percentage. (*F*) Gene synteny plot of upstream and downstream of the photosynthetic gene encoding the homologous protein proteorhodopsin (see also supplementary fig. S8*C*, Supplementary Material online). The left column represents the mycobacterial strain, the locus tag of the first and last genes in the gene synteny plot is represented by the prefix “MCHLDSM_,” “MCHUDSM_,” and “Mycch_” for *Mchlo*DSM, *Mchu*DSM, and *Myc*NBB4, respectively.

Functional classification of annotated genes was done using RAST subsystem classification for each genome as outlined in Materials and Methods. Number of genes in different functional/ subsystem categories was similar in all four mycobacterial strains with the exception of the two categories “photosynthesis” and “metabolism of aromatic compounds” (fig. 5*A*). In a preliminary analysis, 12 genes were annotated in the first category photosynthesis in both *Mchlo*DSM and *Mchu*DSM whereas none was found either in *Myc*NBB4 or in *Mobu*DSM. The 12 genes included multiple copies of genes encoding octaprenyl diphosphate synthase, phytoene dehydrogenase, beta-carotene ketolase, and single copies of genes encoding proteorhodopsin, phytoene synthase, and lycopene beta cyclase. However, in depth analysis based on sequence similarity (see Materials and Methods) revealed that among these 12 genes only the proteorhodopsin gene, which is important for green light absorption, is unique in *Mchlo*DSM and *Mchu*DSM. Both proteorhodopsin genes contain a domain which is 90% identical compared with bac_rhodopsin (bacterial rhodopsin like proteins, AccCdd:smart01021) at the protein level. Genes homologous to the other 11 genes in this category were identified to be present in all these four mycobacterial strains. Moreover, analysis of the gene synteny suggested that the gene encoding proteorhodopsin is missing in *Myc*NBB4 and *Mobu*DSM (fig. 6*F*; see also Discussion).

With respect to the category metabolism of aromatic compounds a near 2-fold higher number of genes were identified in *Mchlo*DSM relative to the other mycobacterial strains. Irrespective of strain these genes encode proteins involved in the degradation of a number of different organic compounds. Here, the most apparent difference among the four strains is that the *Mchlo*DSM type strain harbors several genes encoding proteins that are involved in the central meta-cleavage pathway of aromatic compound degradation (fig. 5*B*). For a compilation of the genes in this category see supplementary table S5, Supplementary Material online. An important class of enzymes in this context is the mono- and dioxygenases (Bugg 2003; Fuchs et al. 2011) and *Mchlo*DSM has the highest number of genes encoding this class of enzymes (fig. 5*C*; see also the Discussion and supplementary fig. S7, Supplementary Material online). These genes were distributed around the *Mchlo*DSM chromosome and this is also the case for the other three *Mycobacterium* spp. (supplementary fig. S1, Supplementary Material online).

Next, we did a functional classification of the unique genes found in all four strains (fig. 5*B*). More than 22% of the total number of nonhomologous genes in the *Myc*NBB4 strain was classified in the category “fatty acids, lipids, and isoprenoids.” For the other three species this value was lower; in particular for *Mchlo*DSM in which less than 5% of the unique genes belonging to this category. Moreover, the *Mchu*DSM strain has only one gene in the functional category “stress response” whereas the others contain several, for example 5% in *Myc*NBB4. Detailed analysis suggested that the unique stress response genes in *Mchlo*DSM are related to oxidative stress whereas in *Myc*NBB4 and *Mobu*DSM they are involved in both oxidative and osmotic stress response (not shown). It should also be noted that none of the unique genes in *Mchu*DSM belongs to the “membrane transport” category, which is not the case for the other strains.

Although these mycobacteria are nonpathogenic it is interesting that all four strains were predicted to have several genes in the “virulence, disease, and defense” category (see also Discussion). Here, *Mchlo*DSM is suggested to have the highest number of unique genes in this category whereas *Mchu*DSM the lowest. The majority of these genes were identified as homologs of *mce* (mammalian cell entry) genes, a class of genes encoding proteins involved in mycobacterial cell invasion and virulence (for a review see [Zhang and Xie 2011]). Moreover, genes encoding proteins with a putative role in copper homeostasis were identified. This was particularly apparent for *Mchlo*DSM in which 28 putative genes were detected on the chromosome (fig. 5*D* and *E*; one was predicted to be located on a plasmid fragment, see supplementary table S1, Supplementary Material online) and many of these genes are clustered near the *oriC* on the *Mchlo*DSM chromosome (supplementary fig. S1*A*, Supplementary Material online).

### Identification of Horizontally Transferred Genes

Next, we predicted the number of horizontally transferred genes on the chromosome in each of the four mycobacterial strains following the criteria as outlined in Materials and Methods (genes carried on plasmids might also be classified as HGT-genes but these were not included here). As shown in figure 6*A* our data suggest that depending on species the number of horizontally transferred genes vary between 220 and 262. Of the predicted HGT-genes 81 were common while the highest number of unique HGT-genes were detected in *Mobu*DSM (fig. 6*B*). Analysis of the GC-content of the HGT-genes in all four strains showed that roughly 25% of the HGT-genes have a lower GC-content compared with that of the total genome for the respective strain consistent with these genes being HGT-genes (figs. 1*B* and 6*C*). We also calculated the codon usage for the predicted HGT-genes and compared it with the codon usage for all CDS (supplementary fig. S9, Supplementary Material online). Here, we detected variations in the codon usage frequencies of translational stop codons irrespective of strain, in particular UAG and UGA. (Note that UGA also codes for selenocysteine in many bacteria [http://trna.bioinf.uni-leipzig.de] and this is also the case for the four strains studied here [supplementary table S3, Supplementary Material online]. However, incorporation of selenocysteine at UGA codons depends on a specific selenocysteine insertion sequence downstream of UGA [Thanbichler and Böck 2001].) Moreover, relative to the other strains *Mobu*DSM appeared to differ most in codon frequency usage comparing predicted HGT-genes and total CDS (supplementary fig. S9, Supplementary Material online). The possible origins of the predicted HGT-genes in the four mycobacterial strains were then identified on the basis on BLAST best hits. As shown in figure 6*D* the results suggested that these HGT-genes might have originated from a large number of bacterial species that belong mainly to the groups *Rhizobiales*, *Pseudomonadales*, *Burkholderiales*, *Solirubrobacteriales*, and *Bacillales*.

Functional classification suggested that the HGT-genes belong to four main categories (including metabolism and degradation for the different categories): 1) amino acid and derivatives; 2) carbohydrates; 3) cofactors, vitamins, prosthetic groups, and pigments; and 4) fatty acids, lipids, and isoprenoids (fig. 6*E*). Interestingly, the genomic region harboring the proteorhodopsin gene was predicted to be horizontally transferred in *Mchlo*DSM (fig. 6*F*). This finding is consistent with that these genes were identified as unique in this species (see above). Moreover, as indicated in supplementary figure S8*B*, Supplementary Material online, the proteorhodopsin gene has also been identified in other Actinobacteria. Comparing the gene synteny for those and *Mchlo*DSM and *Mchu*DSM indicated that within this region it is only the proteorhodopsin gene that is common between these Actinobacteria (supplementary fig. S8*C*, Supplementary Material online).

### Classification of Genes Encoding Small RNAs and Regulatory RNA Motifs

**Fig. 7.—:**
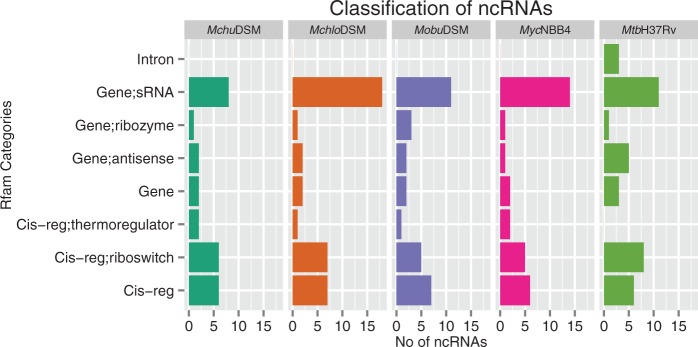
Functional classification of noncoding RNA (ncRNAs). Classification of noncoding RNAs in the *Mchlo*DSM, *Mchu*DSM, *Mobu*DSM, and *Myc*NBB4 strains, for details see the main text.

Like other bacteria *Mchlo*DSM, *Mchu*DSM, *Mobu*DSM, and *Myc*NBB4 do also encode ncRNAs (fig. 1*B*). These ncRNAs were classified and found to belong to different categories based on Rfam (12.0) annotation (fig. 7; supplementary table S6, Supplementary Material online [see also Arnvig and Young 2012]); 1) small RNAs, 2) antisense RNAs, 3) gene; ribozyme, 4) intron, 5) cis-regulatory riboswitches, 6) cis-regulatory thermoregulators, 7) cIS-reg RNAs, and 8) gene. Moreover, compared with the other strains *Mchu*DSM lacks several ncRNAs while *Mchlo*DSM contains several copies of in particular Ms_IGR7. The analysis also revealed unique ncRNAs in *Mchlo*DSM, *Mobu*DSM, and *Myc*NBB4 whereas none was identified in *Mchu*DSM.

## Discussion

We present the genome structure and functional correlation of three mycobacterial species considered to be closely related phylogenetically: the *Mchlo*DSM, *Mchu*DSM, and *Mobu*DSM type strains. Isolates of two of them, *Mchlo* and *Mobu*, show biodegrading properties (Whitman et al. 2012; Satsuma and Masuda 2012; see Introduction). For comparison we included the available complete genome of the *Mycobacterium* strain *Myc*NBB4, which has also been referred to as the *M. chubuense* NBB4 strain. This strain was isolated from the environment on the basis of the presence of genes encoding soluble di-iron monooxygenases. It grows in mineral salt media with ethylene as the sole carbon source and 16S rDNA sequencing positioned *Myc*NBB4 (99% sequence identity) close to *M. chubuense* and *Mycobacterium wolinskyi* (Coleman et al. 2006, 2011; Martin et al. 2014). Phylogenetic analysis based on 16S rDNA, *rpoB*, *dprE*1, and *rnpB* as well as 3,254 core genes present in all four strains and 671 genes present in *Mycobacterium* spp., for which complete genome sequences are available, suggested that *Myc*NBB4 may not be a *M. chubuense* strain. In fact, our data show that the *Mchu*DSM type strain is closer to *Mchlo*DSM than it is to *Myc*NBB4. (Based on comparative analysis of the complete sequence of the 16S rDNA including *M. wolinskyi* it is likely that *Myc*NBB4 is not a *M. wolinskyi* strain [not shown]). Thus, on the basis of our phylogenetic data we suggest that *Myc*NBB4 should be considered as a separate species. Because it was originally isolated on ethylene (Coleman et al. 2006) a suitable name to consider would be *Mycobacterium ethylenense* NBB4. With respect to bacterial speciation we emphasize the difficulties and that high rDNA sequence identity can be misleading because it does not give the total genomic picture (Fraser et al. 2009; Wiedenbeck and Cohan 2011; Rahman et al. 2015).

In this context, we also note that a draft genome of the biodegrading *Mycobacterium rufum* strain JS14 has just been made available (acc no JROA00000000). Phylogeny based on complete 16S rDNA sequences suggested that this *Mycobacterium* spp. is close to *Mchlo*DSM, sharing 99% identity. However, phylogenetic trees generated using *rpoB*, *rnpB*, and *dprE*1 (not shown) as well as core (3111) genes present in *M. rufum* strain JS14, *Mchlo*DSM, *Mchu*DSM, *Mobu*DSM, and *Myc*NBB4 suggested that *Mchu*DSM is closer to *Mchlo*DSM than *M. rufum* strain JS14 (see supplementary fig. S6*F*, Supplementary Material online). Together this emphasizes the importance of using more than one gene for doing phylogenetic analysis (see above). Note also that the genome of *M. rufum* strain JS14 is considerably smaller than that of *Mchlo*DSM, 6.2 versus 6.9 Mb, respectively.

Bioremediation of organic and aromatic pollutants using bacteria such as *Mchlo*DSM and *Myc*NBB4 has attracted much attention. However, plants are the main producers of aromatic compounds in nature but they lack degradation pathways. Microorganisms including those that we studied here play key roles in recycling of carbon derived from aromatic ring structures (Fuchs et al. 2011). Hence, identification of genes encoding enzymes involved in degrading aromatic and organic compounds in various microorganisms is of importance. In this context, both mono- and dioxygenases are key enzymes because they catalyze the oxidation of hydrocarbons by incorporating one and two oxygens, respectively, into the product (Boyd et al. 2003; Bugg 2003). Genes encoding both mono- and dioxygenases were previously identified in *Myc*NBB4 (see e.g., Coleman et al. 2006; Martin et al. 2014 and references therein; see also the annotation of the available *Myc*NBB4 genome). Genes encoding for oxygenases were also identified in the *Mchlo*DSM, *Mchu*DSM, and *Mobu*DSM genomes. For the four strains the number of genes encoding mono-oxygenases exceed the number of dioxygenase encoding genes (fig. 5*C*). Mono- and dioxygenase genes are also found in other *Mycobacterium* spp. such as *M. smegmatis*, *Mycobacterium vanbaalenii* as well as in species that are phylogenetically closely related to these on the basis of 16S rDNA (supplementary figs. S6 and S7, Supplementary Material online). In all these species the total number of oxygenase genes exceeds the number of genes in the four strains studied in this report. However, there is no correlation between the number of genes and genome size. Hence, as we do not have any explanation to this we can only speculate. The mycobacterial species with high number of oxygenase genes might have evolved in environments where these genes provide a growth advantage. In this context we also note that 16S rDNA from the ethene-degrading *Mycobacterium* spp. JS60 show 99% sequence identity compared with that of *Myc*NBB4 and *Mobu*DSM. However, a deeper analysis of the JS60 strain such as the one done in this report is needed to understand its position in the phylogenetic tree relative to *Myc*NBB4 and *Mobu*DSM. We also would like to emphasize that *Nocardia farcinica*, which like *Mycobacterium* belongs to the order Actinomycetales, carries a gene (*nfa*35380 or *rox*) encoding a monooxygenase that renders the bacteria resistant to the front line TB drug rifampicin (Hoshino et al. 2010; Goldstein 2014). Homologs to *nfa*35380 are present in several *Mycobacterium* species (data not shown). Whether the presence and expression of these homologs also result in rifampicin resistance remains to be determined.

Copper (Cu) is required in different biological processes. However, in excess, Cu is toxic (Prohaska and Lukasewycz 1981; Chillappagari et al. 2010; Grass et al. 2011). Recent data suggest that Cu also has some positive effects. Its concentration increases in macrophages upon bacterial infection and higher levels of Cu kills invading bacteria, including *M. tuberculosis*. In order to counter this, many bacteria are equipped with genes encoding enzymes such as multicopper oxidase that allow them to grow inside macrophages (Wolschendorf et al. 2011; Rowland and Niederweis 2013). Add to this the importance of copper-transporting ATPases for copper detoxification in bacteria (White et al. 2009; Shafeeq et al. 2011; Wakeman and Skaar 2012). For *M. tuberculosis* data suggest that copper resistance plays an important role for virulence and CtpV is a copper exporting ATPase needed for full virulence (Ward et al. 2010; Wolschendorf et al. 2011; Wakeman and Skaar 2012; Fu et al. 2014). Hence, it is interesting that 28 genes in the nonpathogenic *Mchlo*DSM were annotated on the chromosome (and one on a plasmid fragment) to encode proteins possibly involved in copper homeostasis and 7 of these were predicted to be horizontally transferred (fig. 5*E*; not shown). The genes were identified as putative copper ATP transporters, copper resistance protein D, multicopper oxidase, and copper chaperone encoding genes (supplementary fig. S1*A*, Supplementary Material online). On the basis of our phylogenetic analysis these 29 genes are suggested to have been acquired after *Mchlo*DSM and *Mchu*DSM diverged. Perhaps this reflects that in the environment where *Mchlo*DSM grows, the presence of these genes confers some selective advantage. For example, such genes would allow growth and survival inside amoebas that engulf bacteria including *Mycobacterium* spp. through phagocytosis (Adékambi and Drancourt 2004; Ben Salah and Drancourt 2010). Nonetheless, we envision that our findings will be useful to understand the evolution of genes involved in copper homeostasis and possibly also with respect to the evolution of efflux pumps and multidrug resistance (Martinez et al. 2009).

The four *Mycobacterium* spp. studied here were isolated from water sediment and soil (see Introduction). In accordance with this many of the predicted horizontally transferred genes are homologous to genes present in other soil and aquatic living bacteria (fig. 6*D*). Interestingly, in both *Mchlo*DSM and *Mchu*DSM we identified homologs to genes encoding proteorhodopsin, light absorbing proteins that act as proton pumps. This gene is also present in *M. rufum* JS14 (acc no JROA01000000) and in some other Actinobacteria (see also Sharma et al. 2006). The gene synteny in both *Mchlo*DSM and *M. rufum* JS14 is similar whereas it is different compared with the other Actinobacteria (supplementary fig. S8*B* and *C*, Supplementary Material online). Proteorhodopsin is the most abundant member of the rhodopsin family and microbial rhodopsin is divided into two categories, transporters and receptors (Sharma et al. 2006). It is found primarily among bacteria living in aquatic environments (Bamann et al. 2014). The proteorhodopsin gene shows high lateral genetic mobility among bacterioplanktons (Frigaard et al. 2006), which is consistent with our data identifying this gene as one of the predicted horizontally transferred genes in two of the mycobacterial strains, *Mchlo*DSM and *Mchu*DSM. Interestingly, the proteorhodopsin gene is positioned close to tRNA genes, which are known to act as targets for integration of foreign DNA into bacterial chromosomes (see e.g., Hacker and Kaper 2000; Juhas et al. 2009). Moreover, it has been suggested that proteorhodopsin influences the response to stress in bacteria (DeLong and Béjà 2010). Whether this also applies to *Mycobacterium* spp. encoding proteorhodopsin and which function rhodopsin has in these mycobacterial species remains to be seen.

Most bacteria only have one gene encoding tRNA^Cys^ (http://trna.bioinf.uni-leipzig.de). However, our analysis showed that *Mchlo*DSM, *Mchu*DSM, and *Mobu*DSM carry an additional tRNA^Cys^ gene, *cysU* (supplementary fig. S3, Supplementary Material online). Further analysis revealed that many of the rapidly growing *Mycobacterium* spp. and some *Rhodococcus* spp. such as *Rhodococcus erythropolis* (but not *Rhodococcus equi*) are also having two tRNA^Cys^ genes (not shown). Putative *cysU* orthologs were also detected in several mycobacterial bacteriophages and in different proteobacteria (not shown). The significance of these findings and why *Mchlo*DSM, *Mchu*DSM, and *Mobu*DSM (and other bacteria) have two tRNA^Cys^ genes is at present not known but it might give a growth advantage. This needs further investigation.

Finally, in the *Mobu*DSM genome we predicted the presence of regions encoding two hammerhead ribozyme structures of class II and III (Birikh et al. 1997; Hammann et al. 2012) similar to that seen in the *M. vanbaalenii* genome (supplementary fig. S10, Supplementary Material online). The putative class II ribozyme is located just at the 3′-end of a gene encoding the alpha subunit of ribonucleotide-diphosphate reductase whereas the other putative hammerhead ribozyme gene is close and positioned at the 3′-end of the neighboring gene, a gene encoding a transcriptional regulator. We also noted that regions encoding putative hammerheads, HH-II and HH-III, are also present in *Mycobacterium intracellulare* but at another location (data not shown). On the basis of the secondary structures of the corresponding hammerhead RNAs we believe that the class III hammerhead might indeed be able to act as a ribozyme. However, this remains to be tested as well as to investigate the biological function of these RNA structures.

## Supplementary Material

Supplementary tables S1–S6 and figures S1–S10 are available at *Genome Biology and Evolution* online (http://www.gbe.oxfordjournals.org/).

Supplementary Data
