# Supplementary material for: Characterization of Three Mycobacterium spp. with Potential Use in Bioremediation by Genome Sequencing and Comparative Genomics
Source: Genome Biol Evol. 2015 Jun 16;7(7):1871–86. doi: 10.1093/gbe/evv111 (PMC4524478; doi:10.1093/gbe/evv111)
Supplement: Supplementary Data [file supp_7_7_1871__index.html]

Characterization of Three Mycobacterium spp. with Potential Use in Bioremediation by Genome Sequencing and Comparative Genomics — Supplementary Data 

# Characterization of Three *Mycobacterium* spp. with Potential Use in Bioremediation by Genome Sequencing and Comparative Genomics

## Supplementary Data

files

- Supplementary Data - pdf file
- Supplementary Data - xlsx file
- Supplementary Data - xlsx file
- Supplementary Data - xlsx file
- Supplementary Data - xls file
- Supplementary Data - xls file
- Supplementary Data - pdf file
- Supplementary Data - pdf file
