# Supplementary material for: Characterization of Three Mycobacterium spp. with Potential Use in Bioremediation by Genome Sequencing and Comparative Genomics
Source: Genome Biol Evol. 2015 Jun 16;7(7):1871–86. doi: 10.1093/gbe/evv111 (PMC4524478; doi:10.1093/gbe/evv111)
Supplement: Supplementary Data [file supp_evv111_Das_et_al_Supplementary_Material_R1.pdf]

Supplementary Material

Table S1 Compilation of contigs belonging to plasmid sequences and their corresponding length and predicted genes in *Mchlo*DSM and *Mobu*DSM.

Table S2 Compilation of genomic coordinates predicted to be prophages in *Mchlo*DSM, *Mchu*DSM, *Mobu*DSM and *Myc*NBB4.

Table S3 Compilation of tRNAs and their corresponding codon in *Mchlo*DSM, *Mchu*DSM, *Mobu*DSM and *Myc*NBB4.

Table S4 Identification and accession number of *Mycobacterium* spp. used in the core gene analysis.

Table S5 Compilation of annotated genes encoding proteins involved in degradation of aromatic and organic compounds.

Table S6 Compilation of ncRNA in *Mchlo*DSM, *Mchu*DSM, *Mobu*DSM and *Myc*NBB4 predicted by Rfam.

Figure S1 Circular representations of the *Mchlo*DSM, *Mchu*DSM, *Mobu*DSM and *Myc*NBB4 genomes illustrating the distribution of tRNA genes and putative genes encoding oxygenases and proteins involved in copper homeostasis

(A) *M. chlorophenolicum* DSM43286

(B) *M. chlorophenolicum* DSM43286 plasmid fragments

(C) *M. obuense* DSM44075

(D) *M. obuense* DSM44075 plasmid fragments

(E) *M. chubuense* DSM44219

(F) *Mycobacterium* spp. NBB4 (*Myc*NBB4)

The outer ring in (A, C, E and F) represents the genome sequence and the green colored ring in (B and D) shows different contigs predicted as plasmid sequences. The altering colors, blue and green, in (A, C, and E) indicate the different contigs while in (F) the complete genome sequence is shown in blue. The red bars represent the positioning of the putative oxygenase genes in all four genomes, while the blue bars together mark the positioning of the different tRNA isoacceptor genes as indicated (* marks the location of the extra tRNA-Cys isoacceptor gene, *cysU*). The black bars (and classification) mark the putative genes encoding proteins in *M. chlorophenolicum* DSM43286 and in *Mycobacterium* spp. NBB4 involved in copper homeostasis. The region highlighted in yellow indicates the clustering of putative copper homeostasis genes in *M. chlorophenolicum* DSM43286 while the blue boxes (one in A and one in E) mark the location of predicted bacteriophage sequences, for details see main text.

Figure S2 Two copies of ribosomal operon in *Mchu*DSM, *Mchlo*DSM and *Mobu*DSM

Track showing average read depth for the ribosomal operon carrying contigs of *Mchu*DSM, *Mchlo*DSM, and *Mobu*DSM. The red boxes mark the genomic region where the rRNA operons are located while the green boxes indicate the read depth of the rRNA operons. Average read depth in the ribosomal operons is approximately double compared to the rest of the genomic regions in the corresponding contigs. The locus tag numbers are indicated below the green boxes. Minimum and maximum values of the average read

depth are indicated on the left hand side of the figure inside third brackets [0-4608], [0-1218] and [0-2663].

Figure S3 Analysis of the genes encoding tRNA^{Cys}

(A) The *cysT* and *cysU* gene synteny for the indicated *Mycobacterium* species. The arrows represent homologous, non-homologous and tRNA^{Cys} genes, *cysT* and *cysU* as indicated. The top part of the figure corresponds to the gene synteny for *cysU* while the bottom for *cysT*. (B) Alignment of the tRNA^{Cys} genes *cysT* (the four sequences at the top) and *cysU* (the three sequences at the bottom) identified in *Mchlo*DSM, *Mchu*DSM, *Mobu*DSM and *Myc*NBB4, however, note that *Myc*NBB4 lacks *cysU*. The "index" MCHLDSM_02154, MOBUDSM_03780 etc refer to the locus tag of the gene in the respective genome.

Figure S4 Heat-map showing Average Nucleotide Identity (ANI) for the four genomes

Genomes are clustered on the basis of the ANI values using hierarchical clustering.

Figure S5 Venn diagrams representing homologous and non-homologous genes by comparing two of the *Mycobacterium* spp. as indicated

Figure S6 Phylogenetic analysis

Phylogenetic trees were generated based on the *rnpB* (A), *rpoB* (B), *dprE*1 (C) 16S rRNA (D), *Mycobacterium* core genes (E) and node genes including *M. rufum* JS14 (F). For details see main text.

Figure S7 Analysis of the presence of mono- and di-oxygenases in different *Mycobacterium* spp.

Distribution of mono- and di-oxygenase in different *Mycobacterium* spp. with respect to their genome size as indicated.

Figure S8 Putative donors of the HGT genes

(A) Complete heat map of the heat map shown in fig. 6 in the main text. Color gradient reflects the number of putative donors from 1 (light) to 30 (dark).

(B) Phylogenetic tree positioning *Mchlo*DSM and *Mchu*DSM close to *Arthrobacter* spp. and other Actinobacteria based on predicted proteorhodopsin genes. The values in percentage represent bootstrap values. (C) Gene synteny plot of the upstream and downstream regions of the predicted proteorhodopsin gene. The left column represents the name of genomes and the locus tag of the first and last genes in the gene synteny plot are shown in the second and third columns, respectively.

Figure S9 Codon usages for horizontally transferred genes and total genes

Relative synonymous codon usages (RSCU) are shown where red refers to total genes and light blue to HGT genes in *Mchlo*DSM (A), *Myc*NBB4 (B), *Mobu*DSM (C) and *Mchu*DSM(D). X-axis represents codons together with their corresponding amino acids. Y-axis represents RSCU.

Figure S10 Presence of putative Hammerhead ribozyme genes in *Mycobacterium* species

Gene synteny for the positioning of the putative hammerhead genes, HHII and HHIII (marked with red arrows) in indicated *Mycobacterium* species. * refers to the gene

encoding the alpha subunit of ribonucleotide-diphosphate reductase while ** refers to a gene encoding a hypothetical protein.
