# Supplementary material for: Characterization of Three Mycobacterium spp. with Potential Use in Bioremediation by Genome Sequencing and Comparative Genomics
Source: Genome Biol Evol. 2015 Jun 16;7(7):1871–86. doi: 10.1093/gbe/evv111 (PMC4524478; doi:10.1093/gbe/evv111)
Supplement: Supplementary Data [file supp_evv111_Supplementary_Table_4_FINAL.pdf]

Supplementary Table 4:
In the current analysis we included 36, *Mycobacterium spp.* complete genomes from NCBI and there reference ids are shown below.

M. abscessus bolletii 50594 NC_021282

M. abscessus NC_010397

M. africanum GM041182 NC_015758

M. avium 104 NC_008595

M. avium subsp. *paratuberculosis* MAP4 NC_021200

M. bovis AF2122 97 NC_002945

M. bovis BCG Pasteur 1173P2 NC_008769

M. bovis BCG Tokyo 172 NC_012207

M. canettii CIPT 140010059 NC_015848

M. chubuense NBB4 NC_018027

M. gilvum PYR GCK NC_009338

M. gilvum Spyr1 NC_014814

M. indicus pranii MTCC_9506 NC_018612

M. intracellulare ATCC 13950 NC_016946

M. intracellulare MOTT 02 NC_016947

M. kansasii ATCC 12478 NC_022663

M. leprae Br4923 NC_011896

M. leprae TN NC_002677

M. liflandii 128FXT NC_020133

M. marinum M NC_010612

M. massiliense GO 06 NC_018150

M. sp. MOTTY36Y NC_017904

M. rhodesiae NBB3 NC_016604

M. smegmatis JS623 NC_019966

M. smegmatis MC2 155 NC_018289

M. smegmatis MC2 155 NC_008596

M. sp. JLS NC_009077

M. sp. KMS NC_008705

M. sp. MCS NC_008146

M. tuberculosis CDC1551 NC_002755

M. tuberculosis F11 NC_009565

M. tuberculosis H37Ra NC_009525

M. tuberculosis H37Rv NC_018143

M. ulcerans Agy99 NC_008611

M. vanbaalenii PYR 1 NC_008726

M. neoaurum VKM Ac 1815D NC_023036

M. yongonense 05 1390 NC_021715
