# Supplementary material for: Characterization of Three Mycobacterium spp. with Potential Use in Bioremediation by Genome Sequencing and Comparative Genomics
Source: Genome Biol Evol. 2015 Jun 16;7(7):1871–86. doi: 10.1093/gbe/evv111 (PMC4524478; doi:10.1093/gbe/evv111)

Supplementary Figure S1:

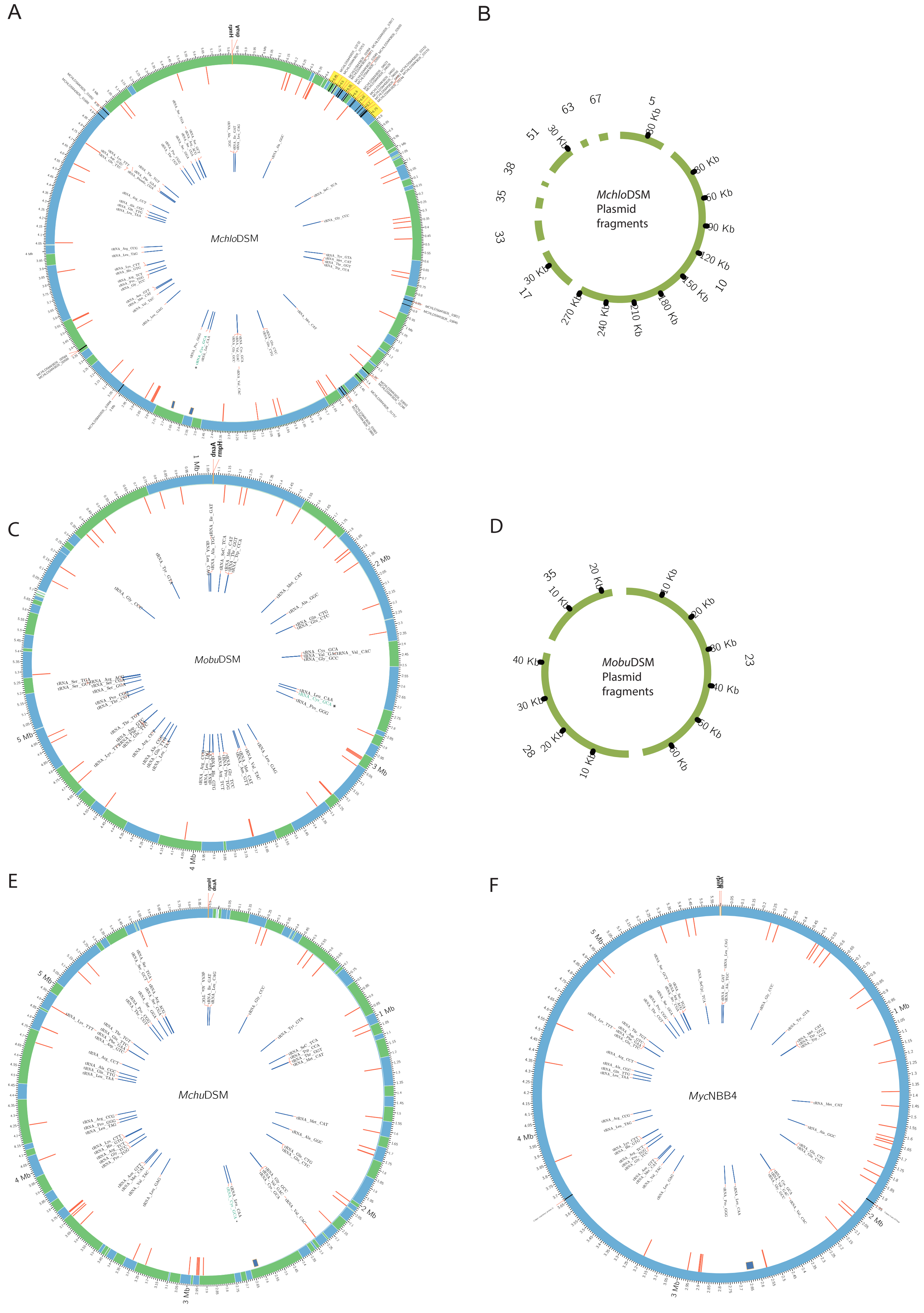

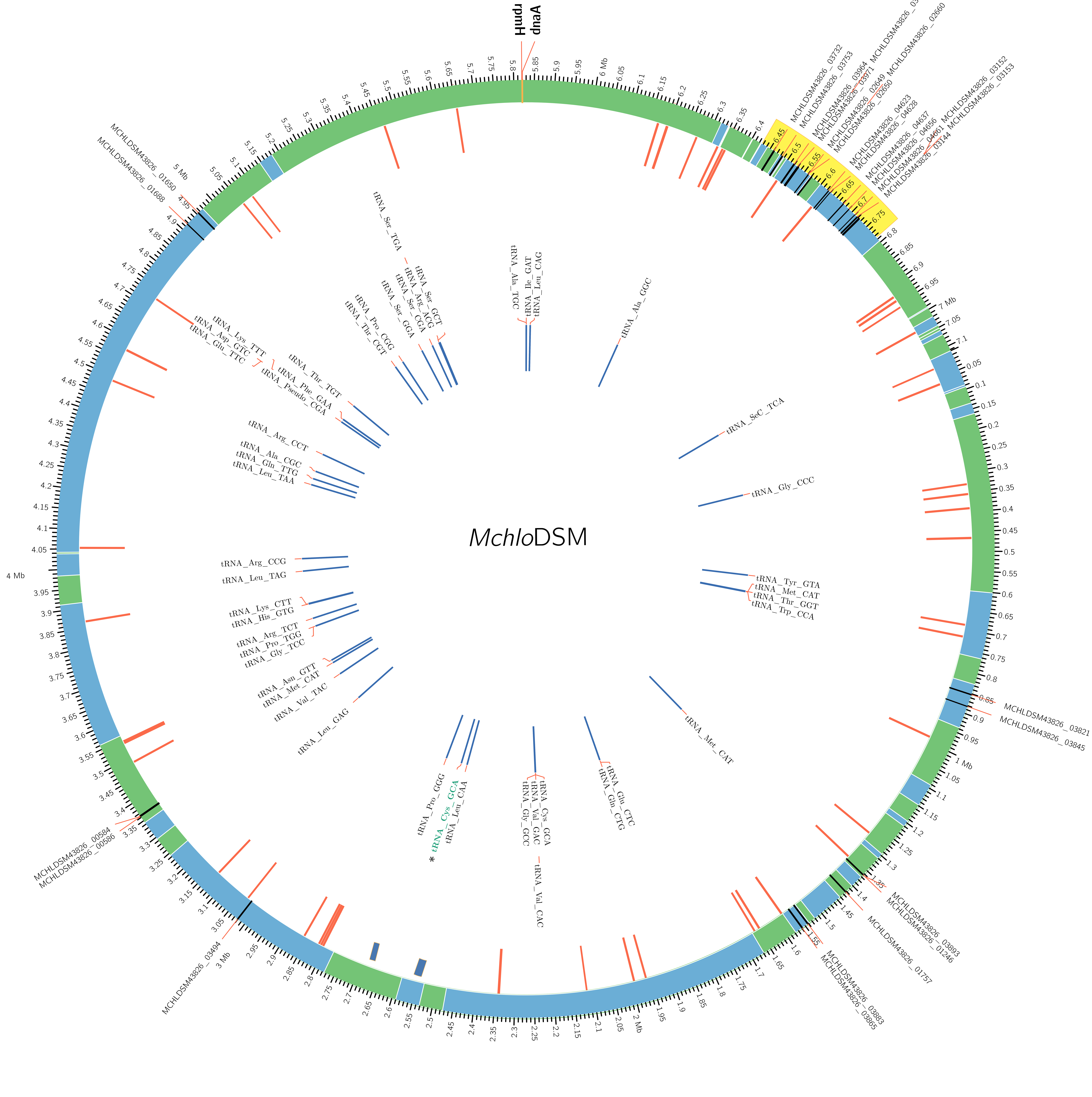

MchloDSM
dnaA
rpmH
MCHLDSM43826_03732
MCHLDSM43826_03753
MCHLDSM43826_03964
MCHLDSM43826_03971
MCHLDSM43826_03972
MCHLDSM43826_02649
MCHLDSM43826_02650
MCHLDSM43826_02660
MCHLDSM43826_04623
MCHLDSM43826_04628
MCHLDSM43826_04637
MCHLDSM43826_04656
MCHLDSM43826_04661
MCHLDSM43826_03144
MCHLDSM43826_03152
MCHLDSM43826_03153
MCHLDSM43826_03821
MCHLDSM43826_03845
MCHLDSM43826_03893
MCHLDSM43826_01246
MCHLDSM43826_01757
MCHLDSM43826_03883
MCHLDSM43826_03865
MCHLDSM43826_03494
MCHLDSM43826_00584
MCHLDSM43826_00586
MCHLDSM43826_01650
MCHLDSM43826_01688
tRNA_Ala_TGC
tRNA_Ile_GAT
tRNA_Leu_CAG
tRNA_Ala_GGC
tRNA_SeC_TCA
tRNA_Gly_CCC
tRNA_Tyr_GTA
tRNA_Met_CAT
tRNA_Thr_GGT
tRNA_Trp_CCA
tRNA_Met_CAT
tRNA_Glu_CTC
tRNA_Gln_CTG
tRNA_Cys_GCA
tRNA_Val_GAC
tRNA_Gly_GCC
tRNA_Val_CAC
tRNA_Pro_GGG
* tRNA_Cys_GCA
tRNA_Leu_CAA
tRNA_Leu_GAG
tRNA_Asn_GTT
tRNA_Met_CAT
tRNA_Val_TAC
tRNA_Arg_TCT
tRNA_Pro_TGG
tRNA_Gly_TCC
tRNA_Lys_CTT
tRNA_His_GTG
tRNA_Arg_CCG
tRNA_Leu_TAG
tRNA_Arg_CCT
tRNA_Ala_CGC
tRNA_Gln_TTG
tRNA_Leu_TAA
tRNA_Thr_TGT
tRNA_Phe_GAA
tRNA_Pseudo_CGA
tRNA_Lys_TTT
tRNA_Asp_GTC
tRNA_Glu_TTC
tRNA_Pro_CGG
tRNA_Thr_CGT
tRNA_Ser_GGA
tRNA_Ser_CGA
tRNA_Arg_ACG
tRNA_Ser_GCT
tRNA_Ser_TGA
6 Mb
7 Mb
1 Mb
2 Mb
3 Mb
4 Mb
5 Mb

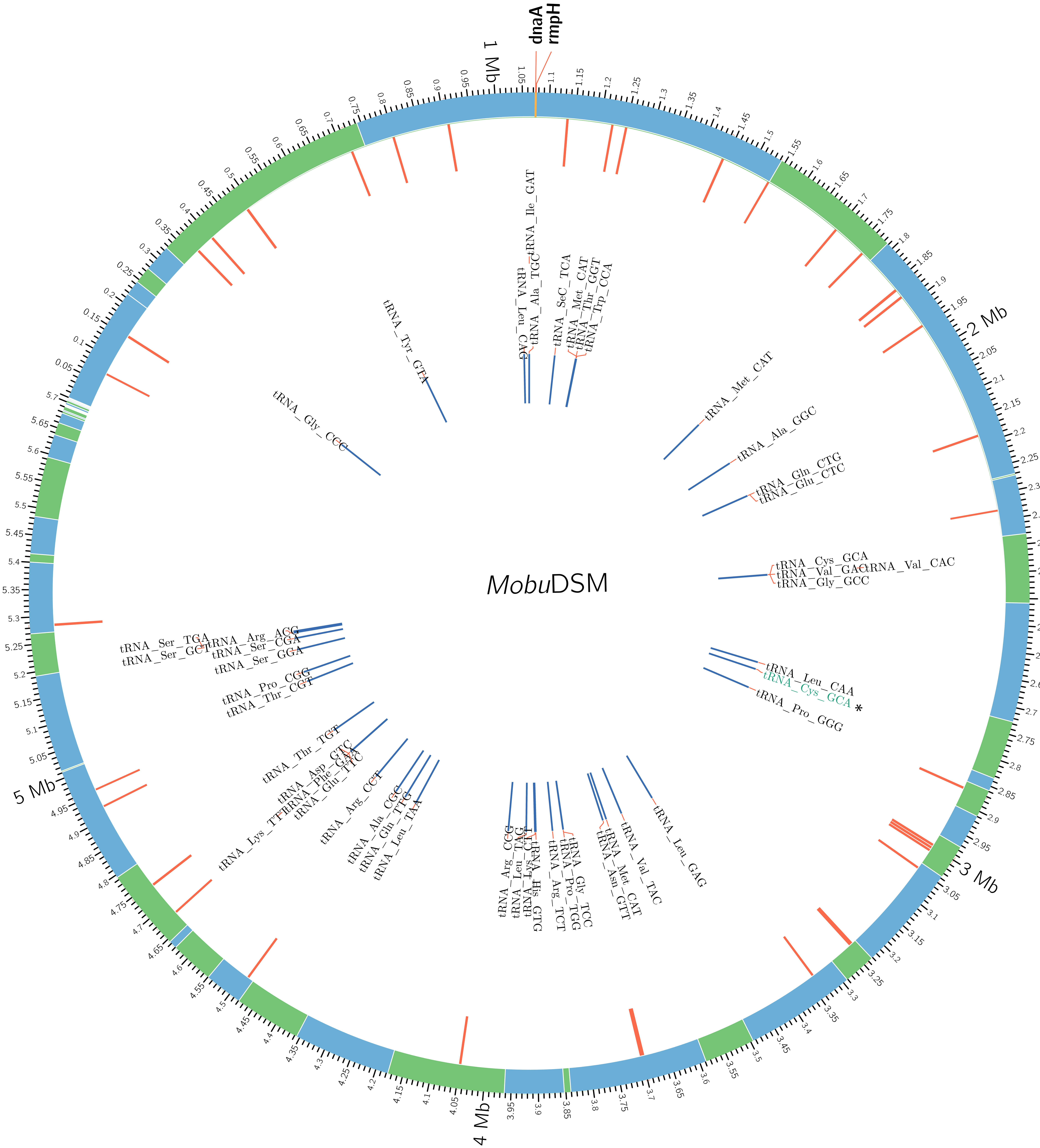

dnaA
rmpH
*Mobu*DSM
1 Mb
2 Mb
3 Mb
4 Mb
5 Mb
tRNA_Ile_GAT
tRNA_Ala_TGC
tRNA_Leu_CAG
tRNA_SeC_TCA
tRNA_Met_CAT
tRNA_Thr_GGT
tRNA_Trp_CCA
tRNA_Tyr_GTA
tRNA_Gly_CCC
tRNA_Met_CAT
tRNA_Ala_GGC
tRNA_Gln_CTG
tRNA_Glu_CTC
tRNA_Cys_GCA
tRNA_Val_GAC
tRNA_Val_CAC
tRNA_Gly_GCC
tRNA_Leu_CAA
tRNA_Cys_GCA *
tRNA_Pro_GGG
tRNA_Leu_GAG
tRNA_Val_TAC
tRNA_Met_CAT
tRNA_Asn_GTT
tRNA_Gly_TCC
tRNA_Pro_TGG
tRNA_Arg_TCT
tRNA_His_GTG
tRNA_Lys_CTT
tRNA_Leu_TAG
tRNA_Arg_CCG
tRNA_Leu_TAA
tRNA_Gln_TTG
tRNA_Ala_CGC
tRNA_Arg_CCT
tRNA_Glu_TTC
tRNA_Phe_GAA
tRNA_Asp_GTC
tRNA_Lys_TTT
tRNA_Thr_TGT
tRNA_Pro_CGG
tRNA_Thr_CGT
tRNA_Ser_TGA
tRNA_Ser_GCT
tRNA_Arg_ACG
tRNA_Ser_CGA
tRNA_Ser_GGA

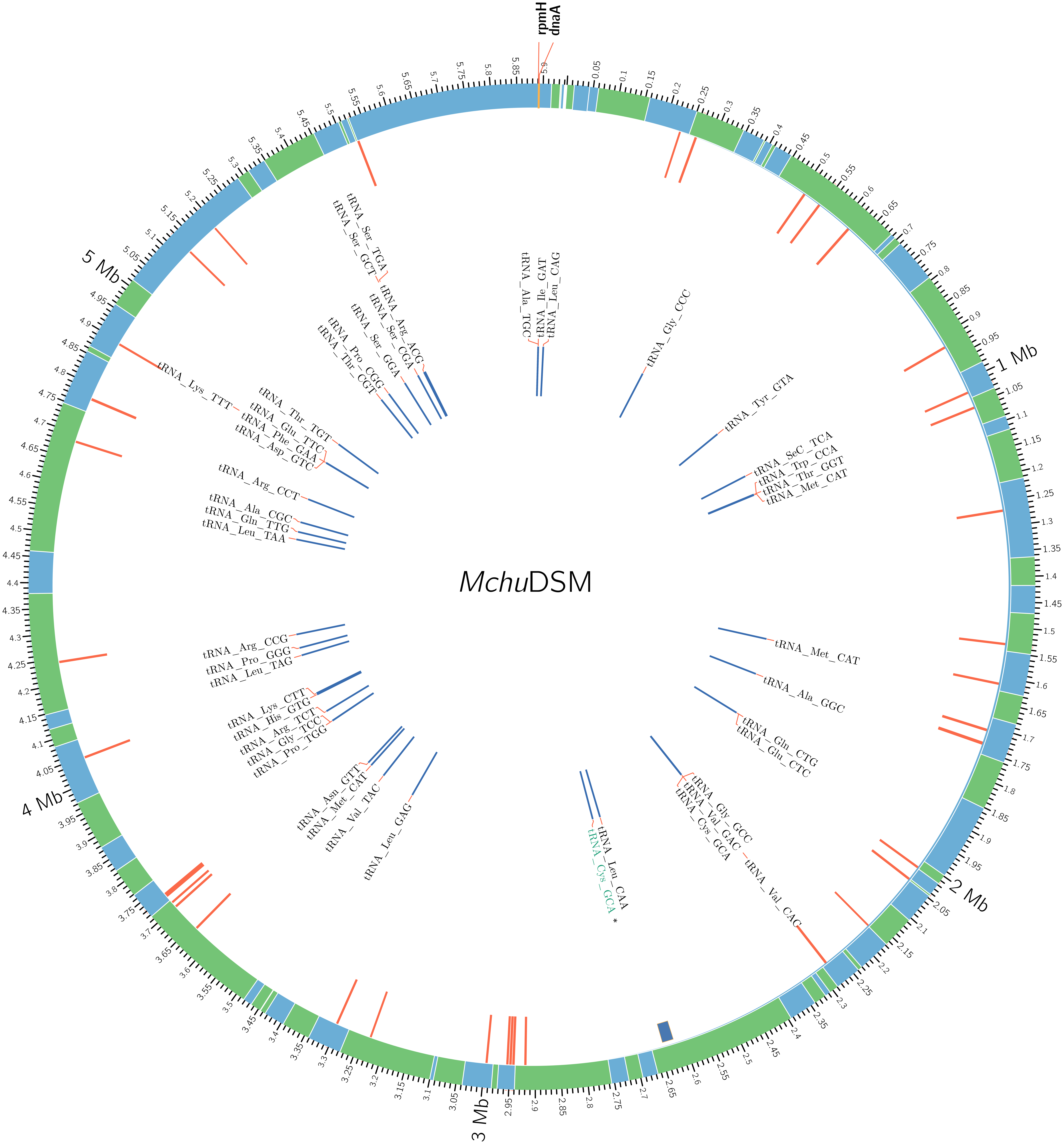
rpmH
dnaA
*Mchu*DSM
1 Mb
2 Mb
3 Mb
4 Mb
5 Mb
tRNA_Ala_TGC
tRNA_Ile_GAT
tRNA_Leu_CAG
tRNA_Gly_CCC
tRNA_Tyr_GTA
tRNA_SeC_TCA
tRNA_Trp_CCA
tRNA_Thr_GGT
tRNA_Met_CAT
tRNA_Met_CAT
tRNA_Ala_GGC
tRNA_Gln_CTG
tRNA_Glu_CTC
tRNA_Gly_GCC
tRNA_Val_GAC
tRNA_Cys_GCA
tRNA_Val_CAC
tRNA_Leu_CAA
tRNA_Cys_GCA
*
tRNA_Leu_GAG
tRNA_Asn_GTT
tRNA_Met_CAT
tRNA_Val_TAC
tRNA_Lys_CTT
tRNA_His_GTG
tRNA_Arg_TCT
tRNA_Gly_TCC
tRNA_Pro_TGG
tRNA_Arg_CCG
tRNA_Pro_GGG
tRNA_Leu_TAG
tRNA_Ala_CGC
tRNA_Gln_TTG
tRNA_Leu_TAA
tRNA_Arg_CCT
tRNA_Thr_TGT
tRNA_Glu_TTC
tRNA_Phe_GAA
tRNA_Asp_GTC
tRNA_Lys_TTT
tRNA_Pro_CGG
tRNA_Thr_CGT
tRNA_Ser_GGA
tRNA_Ser_CGA
tRNA_Arg_ACG
tRNA_Ser_TGA
tRNA_Ser_GCT

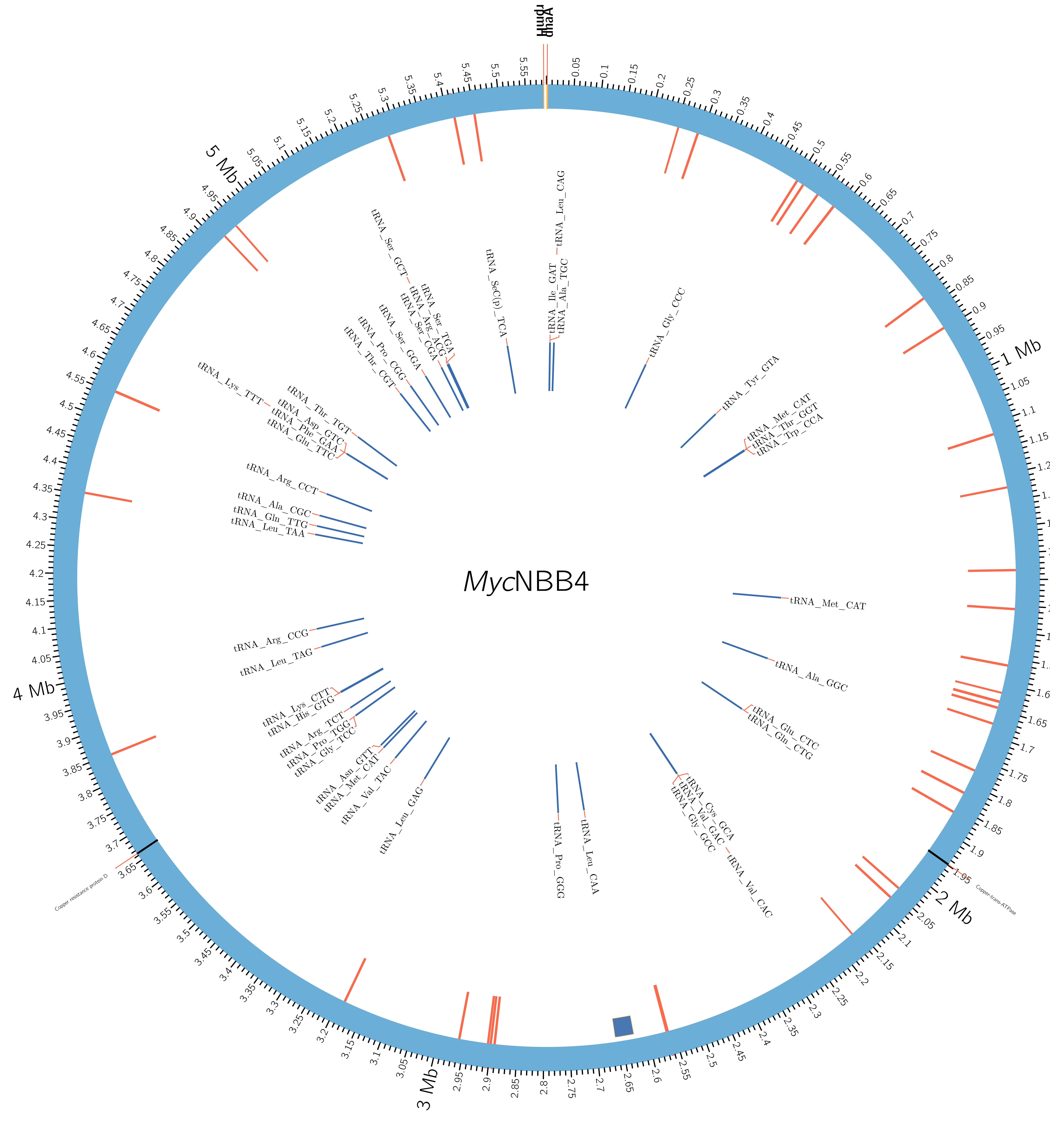

MycNBB4
1 Mb
2 Mb
3 Mb
4 Mb
5 Mb
dhaA
hupJ
Copper resistance protein D
Copper-trans-ATPase
tRNA_Leu_CAG
tRNA_Ile_GAT
tRNA_Ala_TGC
tRNA_SeC(p)_TCA
tRNA_Gly_CCC
tRNA_Tyr_GTA
tRNA_Met_CAT
tRNA_Thr_GGT
tRNA_Trp_CCA
tRNA_Met_CAT
tRNA_Ala_GGC
tRNA_Glu_CTC
tRNA_Gln_CTG
tRNA_Cys_GCA
tRNA_Val_GAC
tRNA_Gly_GCC
tRNA_Val_CAC
tRNA_Leu_CAA
tRNA_Pro_GGG
tRNA_Leu_GAG
tRNA_Val_TAC
tRNA_Asn_GTT
tRNA_Met_CAT
tRNA_Arg_TCT
tRNA_Pro_TGG
tRNA_Gly_TCC
tRNA_Lys_CTT
tRNA_His_GTG
tRNA_Leu_TAG
tRNA_Arg_CCG
tRNA_Ala_CGC
tRNA_Gln_TTG
tRNA_Leu_TAA
tRNA_Arg_CCT
tRNA_Lys_TTT
tRNA_Thr_TGT
tRNA_Asp_GTC
tRNA_Phe_GAA
tRNA_Glu_TTC
tRNA_Thr_CGT
tRNA_Pro_CGG
tRNA_Ser_GGA
tRNA_Ser_GCT
tRNA_Ser_TGA
tRNA_Arg_ACG
tRNA_Ser_CGA

Supplementary Figure S2:

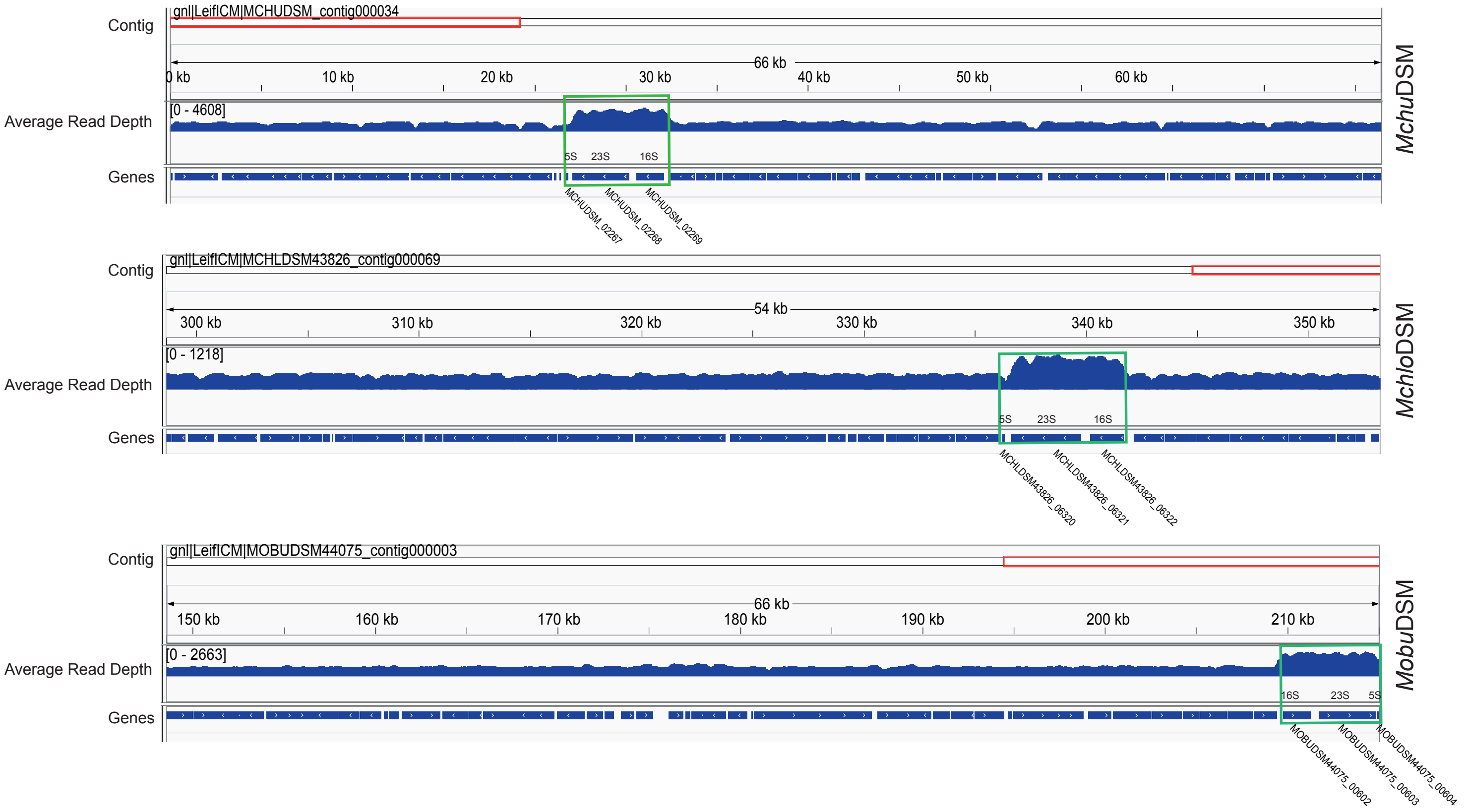

Supplementary Figure S3:

A

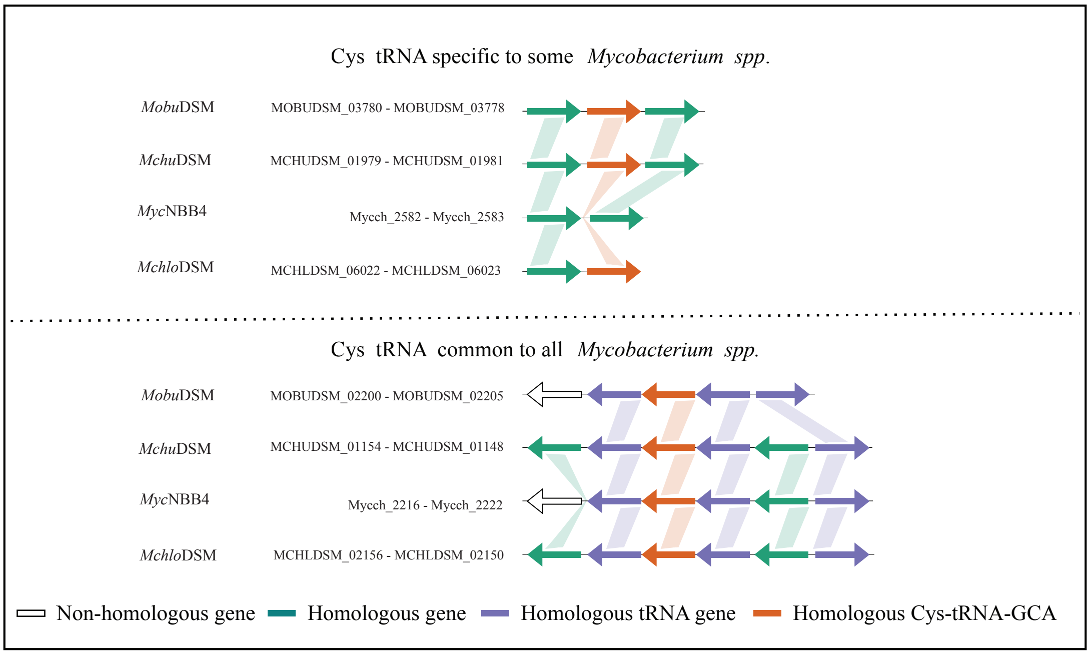

B

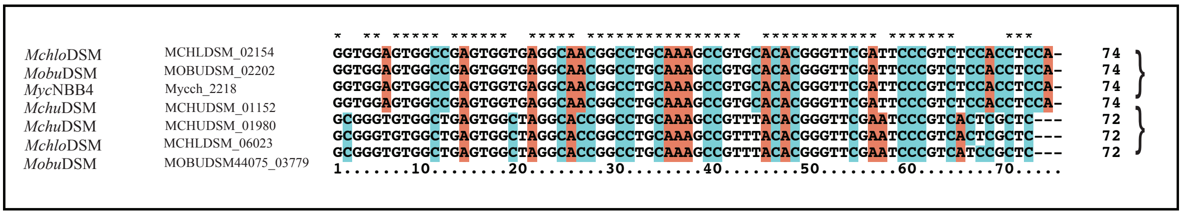

Supplementary Figure S4:

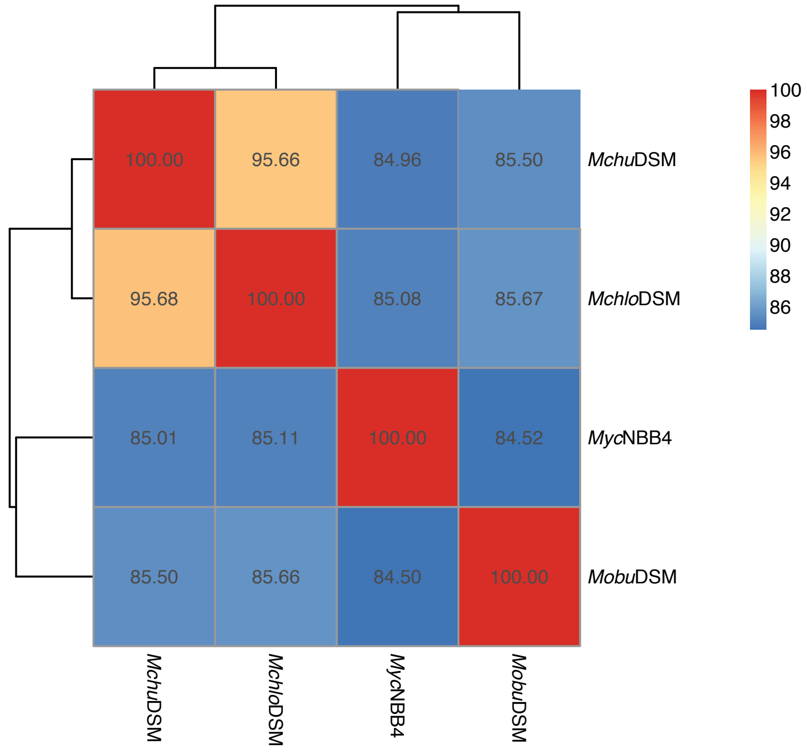

Supplementary Figure S4:

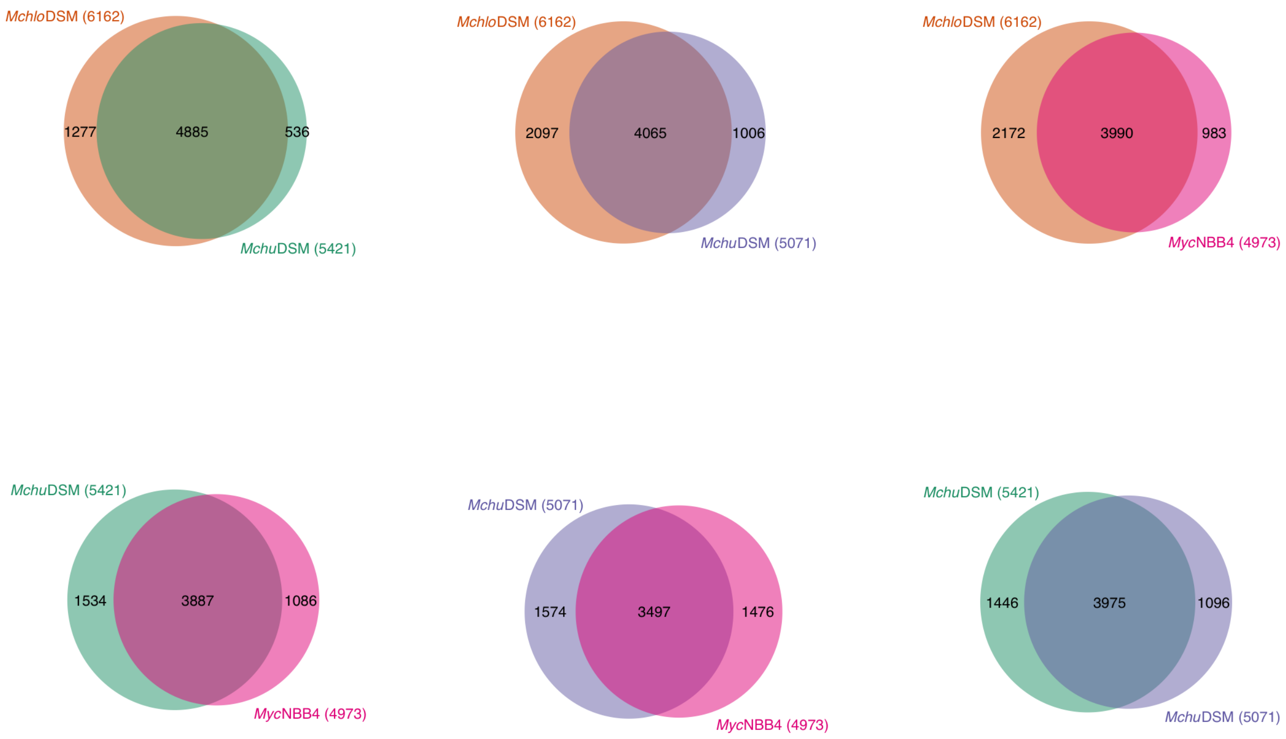

Supplementary Figure S6:

A

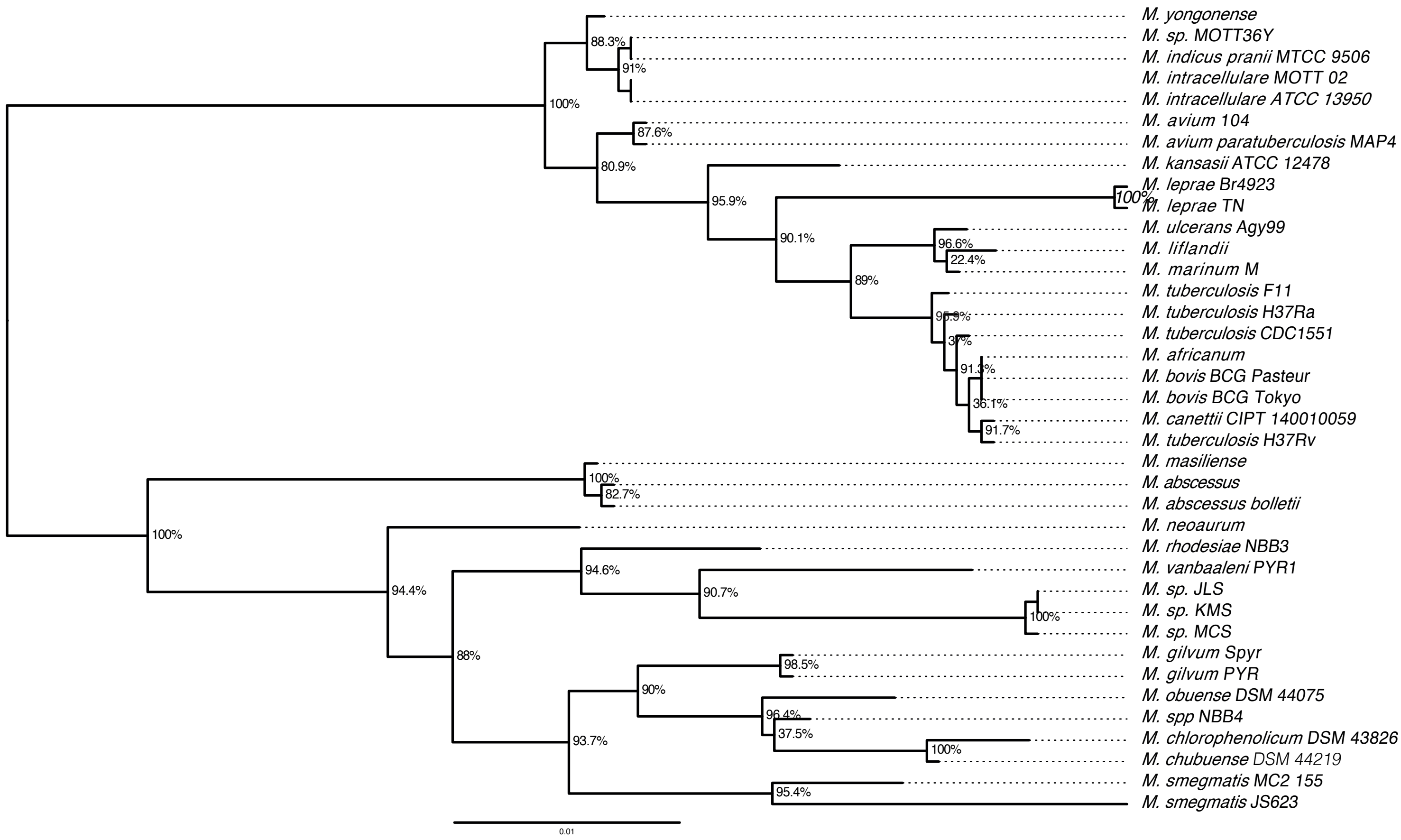

B

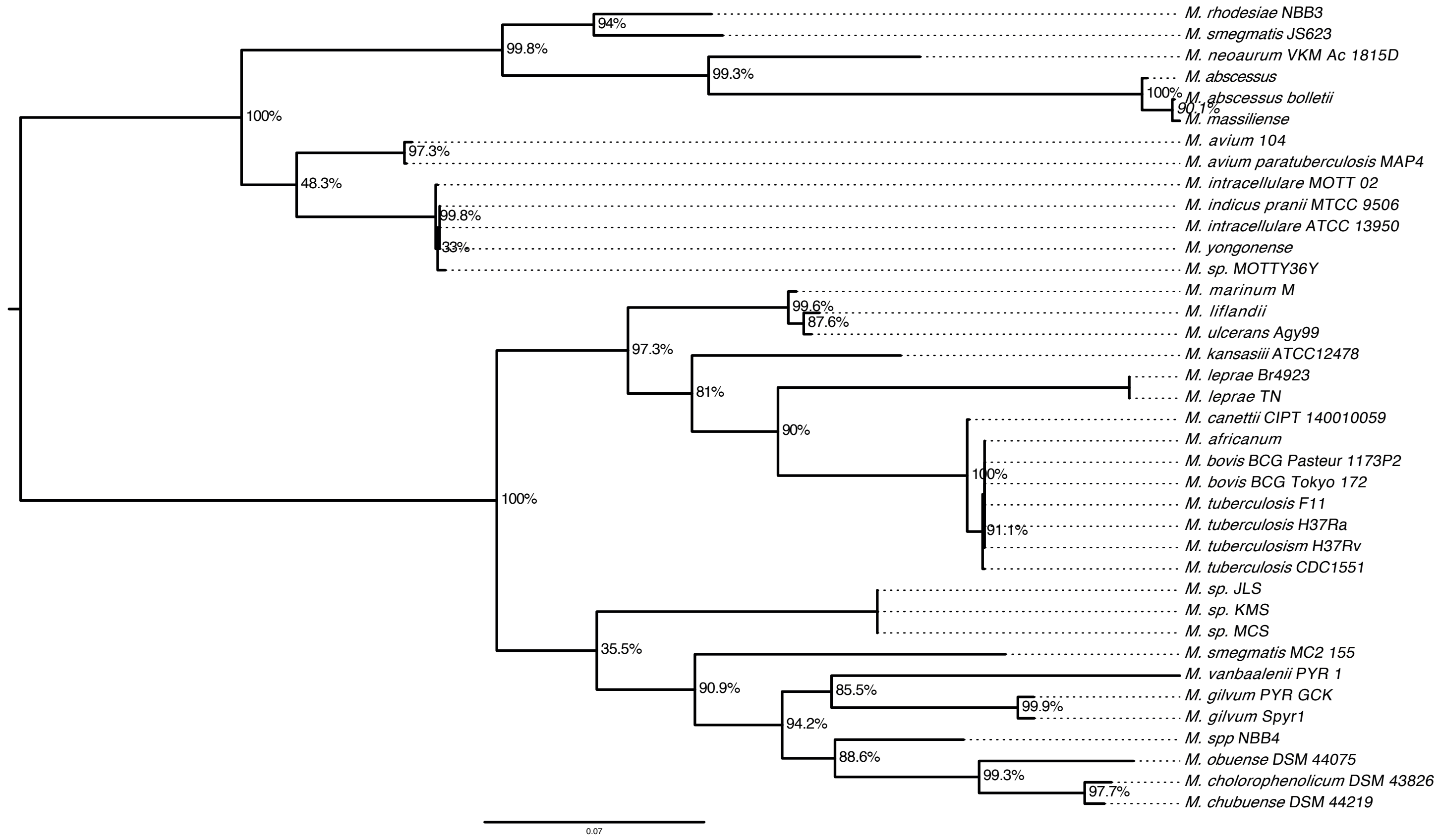

Supplementary Figure S6:

C

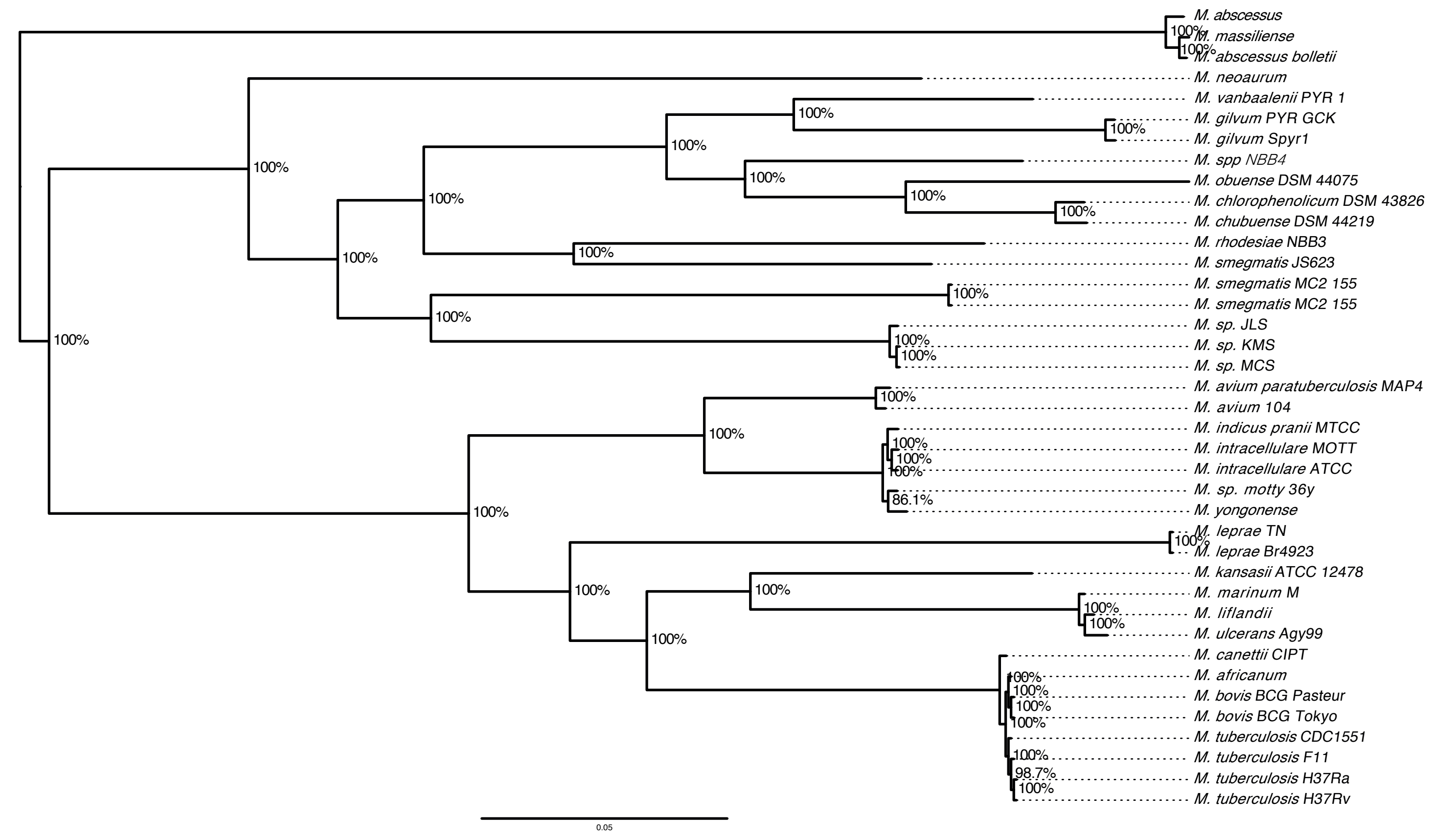

D

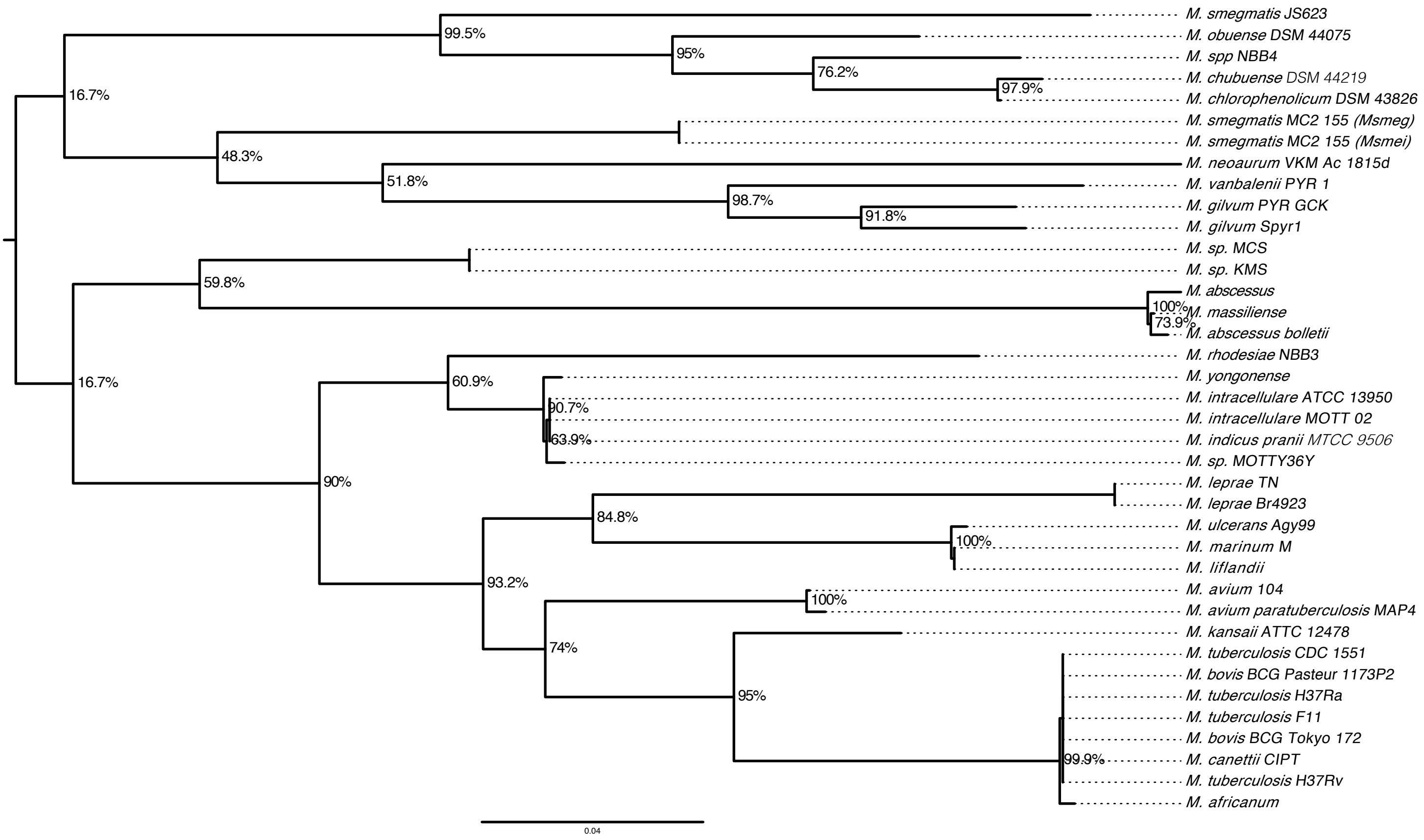

Supplementary Figure S6:

E

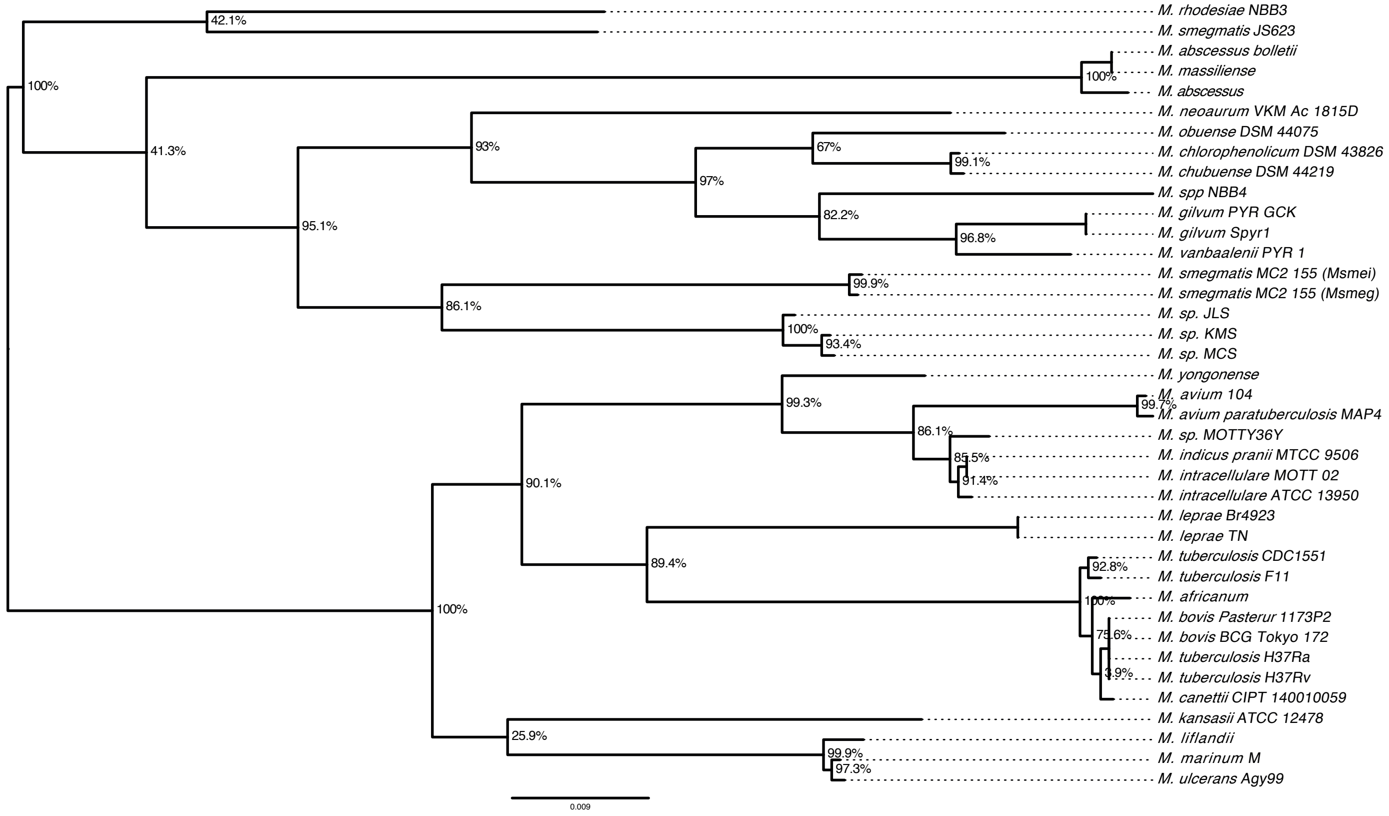

F

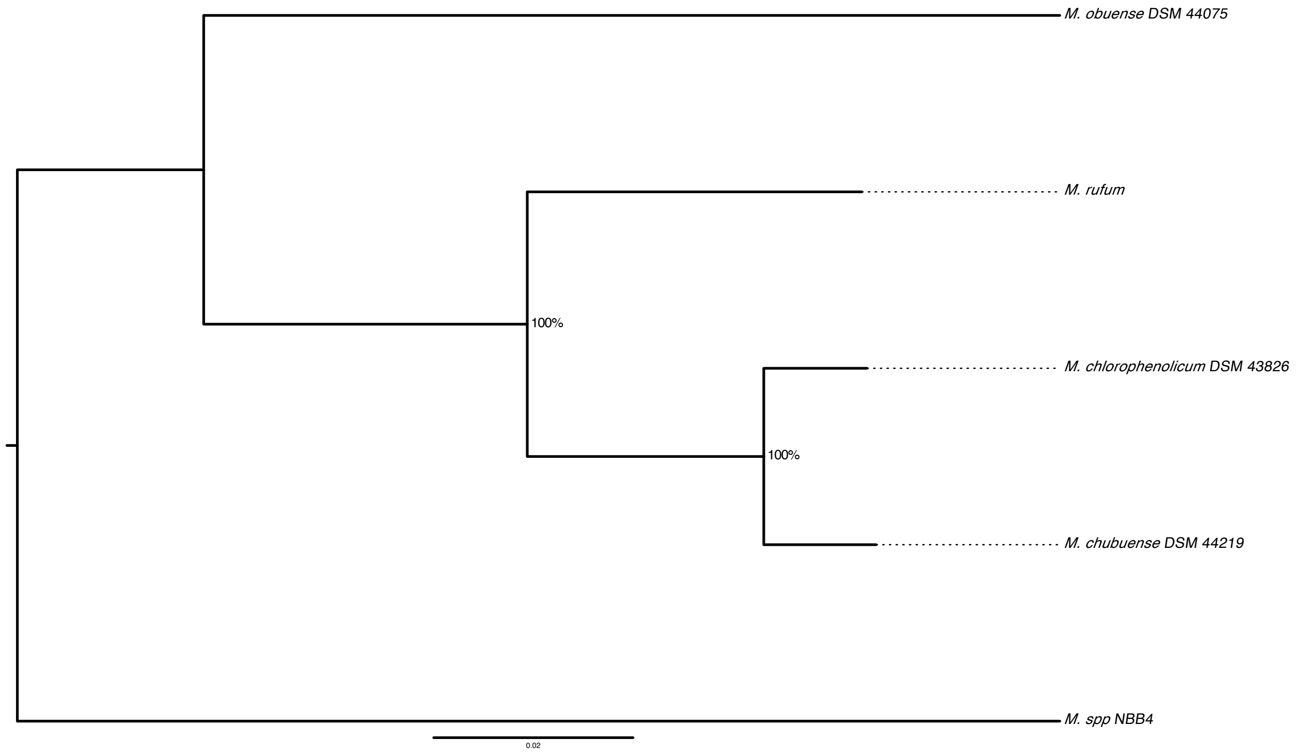

Supplementary Figure S7:

A

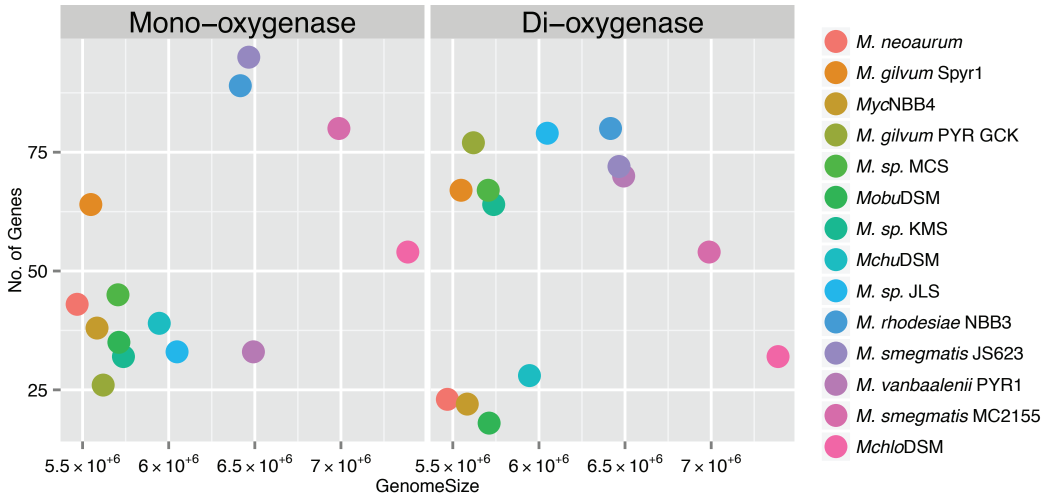

Supplementary Figure S8:

A

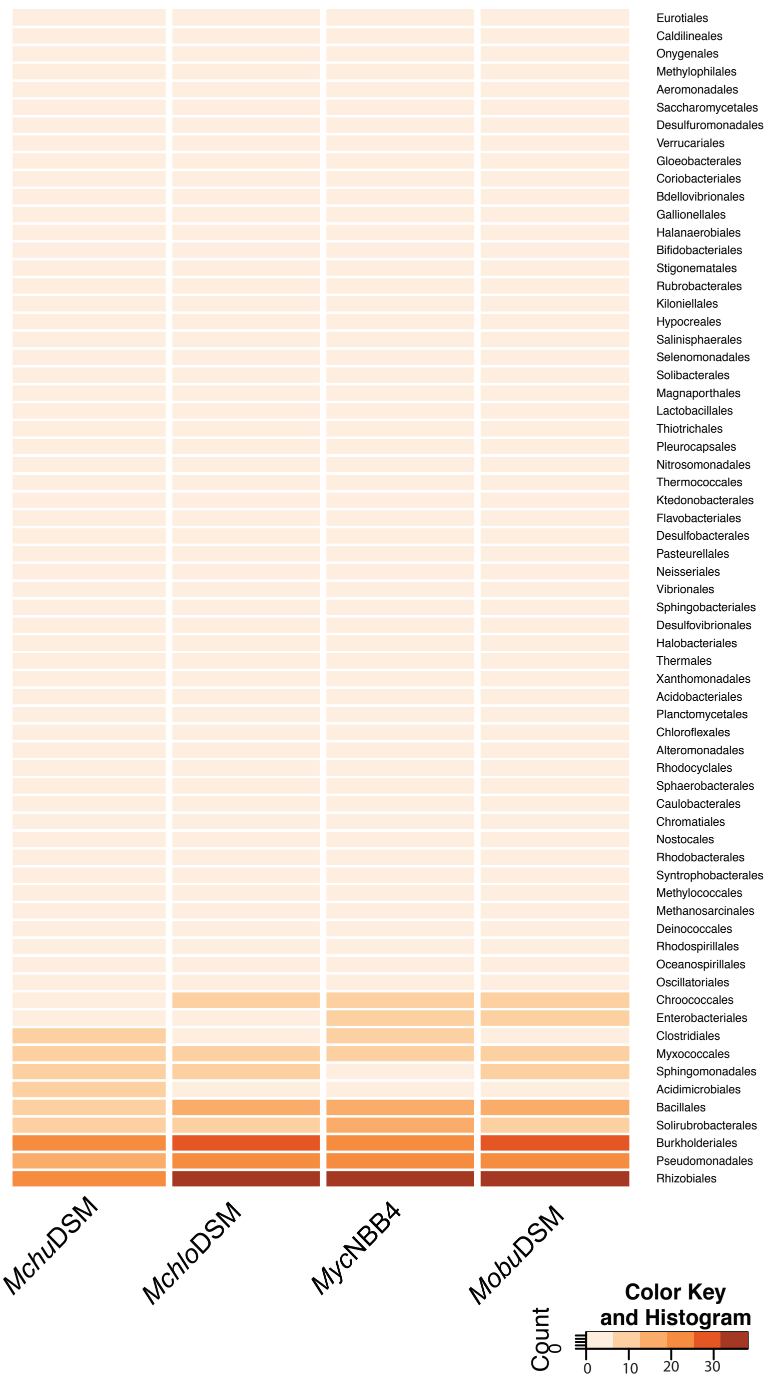

B

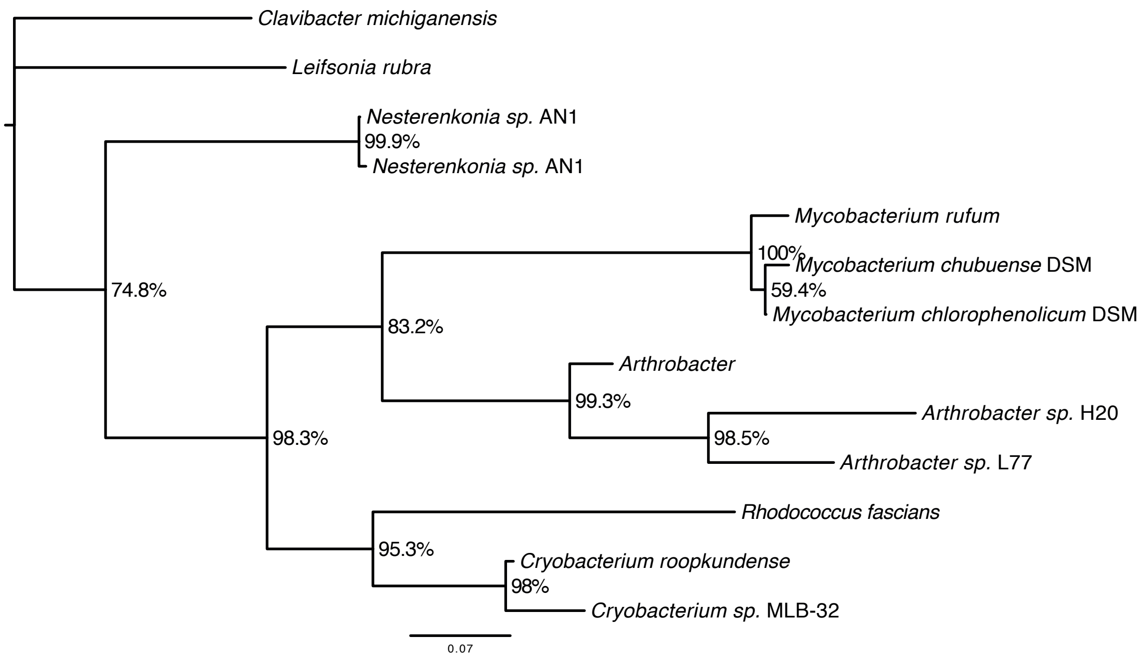

Supplementary Figure S8:

C

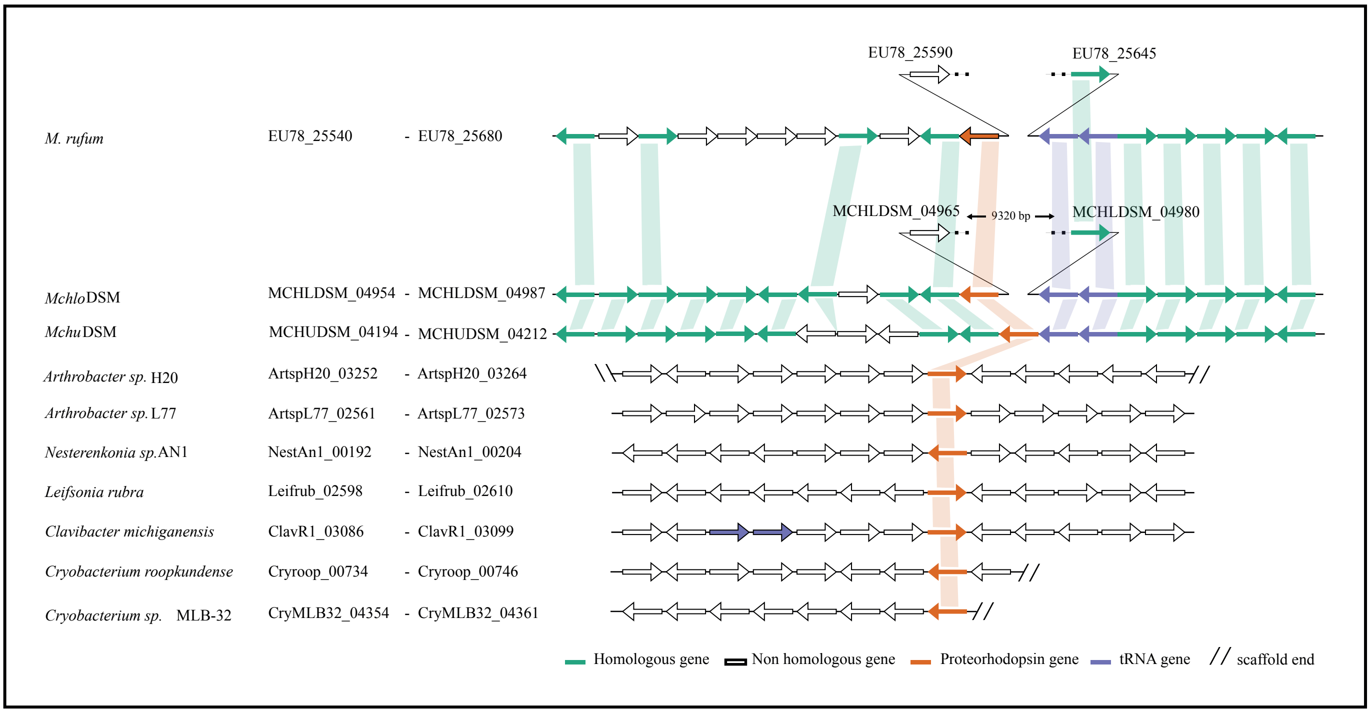

Supplementary Figure S9:

A

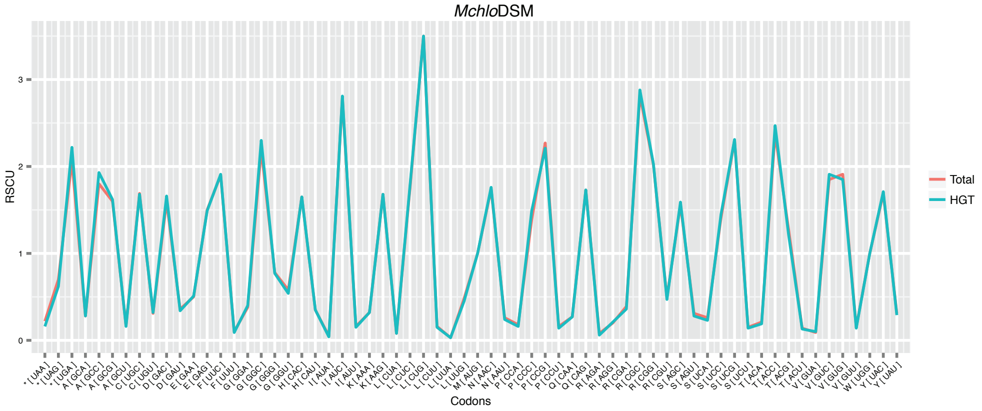

B

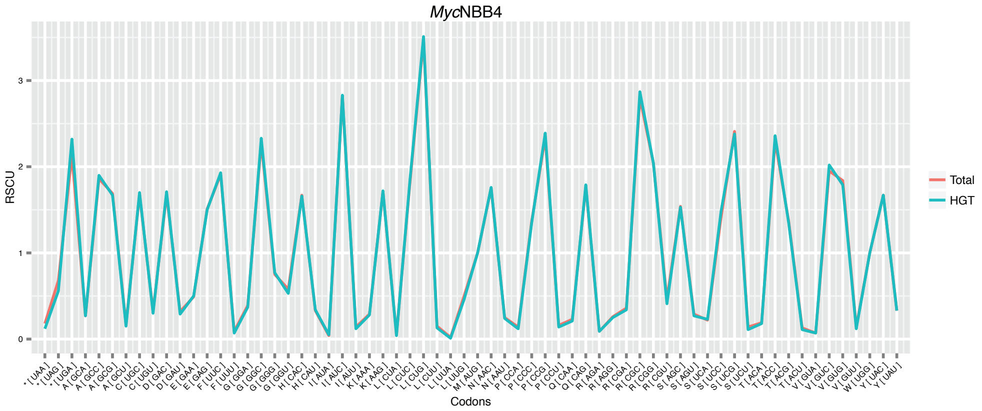

C

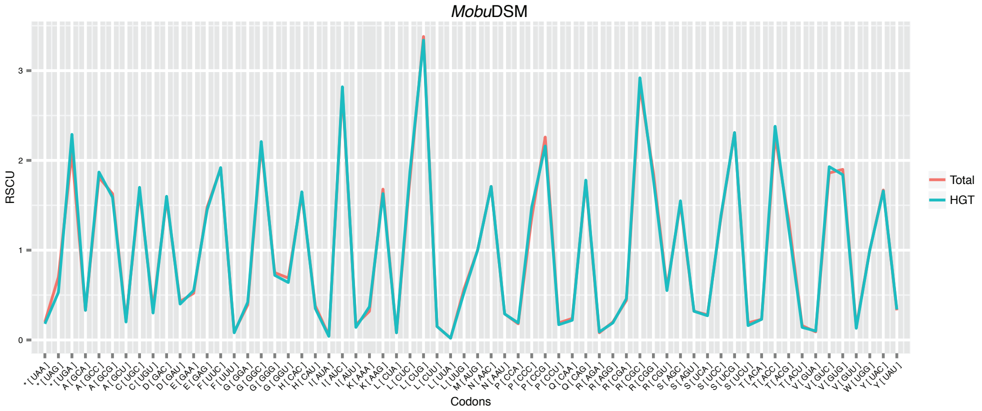

D

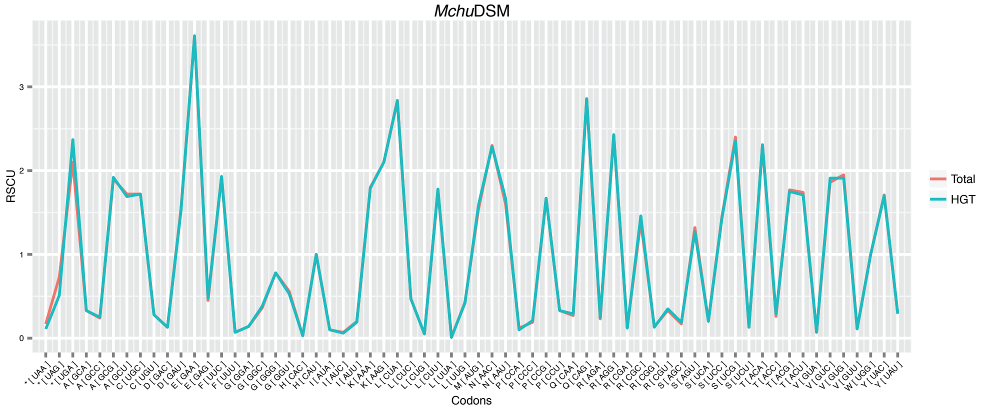

Supplementary Figure S10:

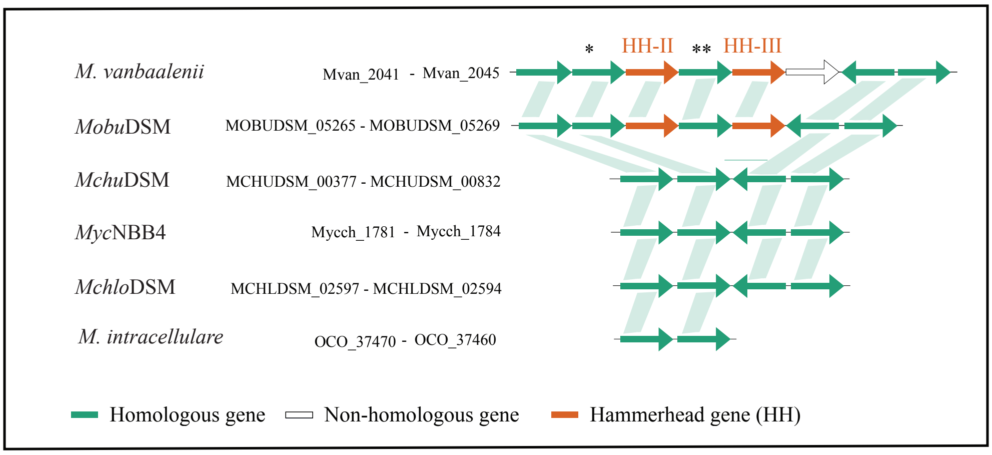

Supplement: Supplementary Data [file supp_evv111_SUPPLENTARY_FIGs_S1-10.pdf]
